# Identification of Potent siRNA Delivery Peptides Using Computer Modeling

**DOI:** 10.1002/advs.202308345

**Published:** 2024-02-04

**Authors:** Ke Men, Mohan Liu, Xueyan Zhang, Yuling Yang, Rui Zhang, Yusi Wang, Die Hu, Bailing Zhou, Li Yang

**Affiliations:** ^1^ Department of Biotherapy, Cancer Center and State Key Laboratory of Biotherapy West China Hospital Sichuan University Chengdu 610041 P. R. China

**Keywords:** computer modeling, gene delivery, nanoparticle, peptide, siRNA

## Abstract

Peptides with suitable aggregation behavior and electrical properties are potential siRNA delivery vectors. However, identifying suitable peptides with ideal delivery and safety features is difficult owing to the variations in amino acid sequences. Here, a holistic program based on computer modeling and single‐cell RNA sequencing (scRNA‐seq) is used to identify ideal siRNA delivery peptides. Stage one of this program consists of a sequential screening process for candidates with ideal assembly and delivery ability; stage two is a cell subtype‐level analysis program that screens for high in vivo tissue safety. The leading candidate peptide selected from a library containing 12 amino acids showed strong lung‐targeted siRNA delivery capacity after hydrophobic modification. Systemic administration of these compounds caused the least damage to liver and lung tissues and has little impact on macrophage and neutrophil numbers. By loading STAT3 siRNA, strong anticancer effects are achieved in multiple models, including patient‐derived xenografts (PDX). This screening procedure may facilitate the development of peptide‐based RNA interference (RNAi) therapeutics.

## Graphical abstract

A systemic program based on computer modeling and single cell RNA‐sequencing is established to identify potent siRNA delivery peptides from library. This program consists of a sequential screening process for candidates with ideal assembly and delivery ability as stage one, and followed by a cell subtype level analyzing for high in vivo tissue safety as stage two.

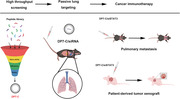

## 1 Introduction

Small interfering RNA (siRNA) therapeutics have exhibited promising efficacy in cancer treatment through gene interference.^[^ ^1^ ^]^ The delivery system is one of the main technical limitations of siRNA drug development.^[^ ^1^, ^2^, ^3^ ^]^ Compared with traditional vectors, several new‐generation siRNA delivery systems with more satisfactory in vivo safety and greater manufacturing convenience have been applied in recent years.^[^ ^2^, ^4^ ^]^ Peptides are fundamental cellular components and are widely involved in bioprocesses.^[^ ^5^ ^]^ Owing to the numerous types of amino acids, their diverse electronic characteristics can enable nucleic acid binding.^[^ ^6^ ^]^ Therefore, peptides and their derivatives with suitable aggregation behavior and electrical properties might be potential vectors for siRNA delivery. Compared to other nonviral gene vectors, peptide vectors share several advantages.^[^ ^7^, ^8^ ^]^ However, the properties of peptide vectors used as gene delivery systems are largely influenced by the amino acids sequence that constitutes the linear structure.^[^ ^8^, ^9^ ^]^ A peptide must be synthesized and purified before its viability can be fully determined. This process is time‐consuming and expensive. Thus, if a prediction method is applied, the development efficiency is expected to be greatly elevated.

Computer‐aided modeling has been widely applied in drug exploration, including structure prediction, molecular dynamics simulation, and binding free energy calculation,^[^ ^10^, ^11^ ^]^ providing an advanced strategy for predicting the chemical and biological properties of candidate compounds. This technology has been successfully applied in the design and screening of chemical drugs before synthesis, resulting in high industrial efficiency.^[^ ^12^ ^]^ Similarly, the dissolving process of peptides largely depends on their distribution and aggregation behaviors in aqueous solution.^[^ ^13^ ^]^ The folding and exposure of certain residues on the surface of peptides is also a dynamic process. In this regard, computer‐aided modeling might also be helpful. In recent years, efforts have been made to study the physicochemical functions of peptides using computer modeling.^[^ ^14^, ^15^ ^]^ However, predicting the siRNA delivery properties of different peptides with diverse sequences is much more difficult. We need to know the details of aggregation to predict the resulting size and shape through computer‐aided modeling.^[^ ^16^ ^]^ It is necessary to understand the dynamic behavior of peptides to evaluate their stability in aqueous solution.^[^ ^17^ ^]^ The surface charge properties must also be understood to predict the potential siRNA binding ability and cytotoxicity of these materials.^[^ ^18^ ^]^ Moreover, this potential computer‐aided modeling method should be able to process many peptides simultaneously, providing accurate comparison data for high‐throughput pre‐synthesis screening.^[^ ^19^ ^]^ An ideal computer‐aided modeling strategy with appropriate parameters will certainly enhance the development efficiency of peptide‐based siRNA vectors.

In vivo safety is another principal requirement of an ideal gene delivery vector, and these vectors are generally delivered via intravenous (i.v.) administration.^[^ ^20^ ^]^ Cell toxicity resulting from positively charged amino acids is a common challenge.^[^ ^21^ ^]^ Cationic agents are rapidly distributed to organ tissues throughout the body, and the positively charged amino acids that support the peptide's gene delivery capacity, leading to safety concerns. In addition, when comparing two peptides with similar delivery abilities, it is difficult to distinguish their exact difference at the cellular level. Moreover, with MTT and hematoxylin and eosin (HE) staining, the molecular mechanisms that regulate cytotoxicity cannot be elucidated, thus failing to provide evidence for “structure‐safety”‐directed development. Therefore, a more detailed analysis method with higher resolution and greater analysis depth may be necessary. Here, we report a computer‐aided modeling‐based high‐throughput strategy for the pre‐synthesis prediction and screening of ideal peptide‐based gene vectors from a library. Moreover, single‐cell RNA‐seq analysis was utilized to investigate the in vivo safety mechanisms after i.v. administration. The qualified peptide vector was further evaluated for siRNA‐based cancer gene therapy in multiple models.

## 2 Results

### 2.1 Computer Modeling‐Based Screening of a Peptide Library

**Figure 1:**
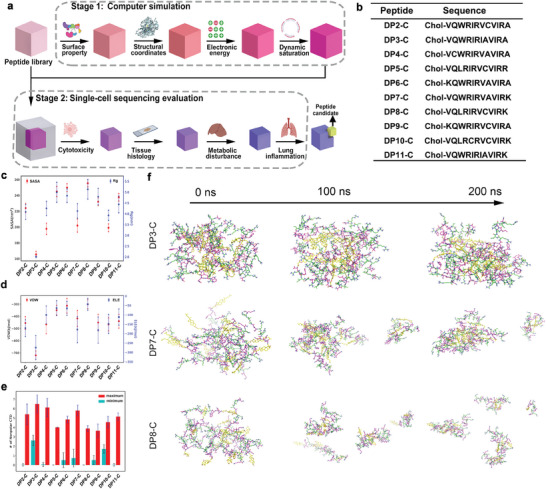
Overview of the techniques presented in this study for analyzing peptide libraries. a) The process of peptide construction, selection, and ranking by rational design. b) Sequences of 10 peptide‐cholesterol conjugates. c) The total SASA and Rg values of different cholesterol‐peptide systems in the simulations (red: SASA; blue, Rg). d) The noncovalent binding energies VDW and ELE of all cholesterol–peptide systems in the simulations (red: VDW; blue, ELE). e) The maximum and minimum number of hydrophobic (CTD) regions around the core of the nanoparticles (red: maximum; green, minimum). f) The representation of different peptide aggregations after the molecular dynamic simulations. The carbons of the hydrophobic terminals are shown in yellow on the left. The carbons of the changeable residues are in magenta, while those of the conserved residues are shown in green.

To achieve high‐throughput screening of optimal candidates in the peptide library, we established a computer‐modeling design strategy to predict the characteristics of candidates, such as size, hydrophobicity, and conformational propensities (**Figure** **1**a). Herein, ten peptides were selected as candidates (Figure 1b). These peptides were randomly designed and were all composed of 12‐mer amino acids. Our computer modeling design strategy was applied to these candidates to predict the optimal peptide sequences (Figure 1a,b). To evaluate the aggregation process of different peptides, the solvent‐accessible surface area (SASA) and the radius of gyration (Rg) were calculated for different peptides.^[^ ^22^, ^23^ ^]^ The SASA is a variable that describes the surface area of a molecule that is accessible to the solvent, and the Rg is another important property of the structure that reflects the shape of a molecule.^[^ ^22^, ^23^ ^]^ As shown in Figure 1c, the DP3‐C peptide had the smallest SASA value (165.48±3.33), which indicated that it had the highest degree of aggregation (Figure 1c). In addition, DP6‐C (240.04±13.01) and DP8‐C (252.18±6.66) had the largest SASA values (Figure 1c), suggesting that they exhibited the lowest aggregation (Figure S1, Supporting Information). As expected, the Rg values represented by blocks exhibited a trend similar to that of the SASA values (Figure 1c). To better understand the binding of these peptides to solvents, the vacuum potential energy, which mainly includes electrostatic (ELE) interactions and van der Waals (VDW) interactions, was assessed.^[^ ^24^, ^25^ ^]^ According to our results, the DP3‐C peptide had the strongest VDW (−720.32±32.86) and ELE (−274.84±62.37) binding energies, indicating the highest aggregation, which was consistent with its SASA and Rg results, while those of DP7‐C (VDW: −416.77±62.41; ELE: −178.41±68.03) and DP10‐C (VDW: −466.31±50.82; ELE: −148.42±49.24) were comparatively lower (Figure 1d). Additionally, the ELE binding energies of DP5‐C (−64.33±38.03), DP6‐C (−65.89±35.82), and DP8‐C (−43.74±29.83) barely contributed to peptide binding due to the presence of too many positively charged residues. Additionally, the VDW interactions in DP3‐C, DP7‐C and DP10‐C were much stronger than those in the other peptides (Figure 1d). Since peptide aggregation relies on the nonpolar carbon terminal domain (CTD), we then defined a variable, named the saturation number, which indicates the number of nonpolar CTD parts approximately 3 Å from the selected CTD.^[^ ^26^ ^]^ DP3‐C had the largest average maximum number of surrounding CTDs (−43.74±29.83), indicating the greatest aggregation (Figure 1e). Among these 10 peptides, DP4‐C and DP6‐C had the second highest saturation numbers (Figure 1e). All of these binding energy analyses showed that DP3‐C, DP7‐C and DP10‐C easily aggregated. We then investigated the detailed dynamic process of peptide aggregation at different simulation times. As shown in Figure 1f and Figure S1 (Supporting Information), DP3‐C, DP4‐C, DP6‐C, DP7‐C, DP10‐C, and DP11‐C quickly aggregated together in the first 10 ns, while DP6‐C and DP11‐C gradually failed to form a sphere‐like structure within 200 ns. In contrast, DP3‐C, DP4‐C, DP7‐C and DP10‐C were clustered at every point. DP2‐C, DP5‐C, DP8‐C, and DP9‐C did not aggregate after 200 ns.

From the binding energy values, we predicted that peptides with too many positively charged residues (such as DP5‐C, DP6‐C, and DP8‐C) would reduce peptide binding. In addition, the aggregation status suggested that the aromatic indole can bind to the hydrophobic (nonpolar CTD) region of the peptides, which can further help the aggregation of the peptides (such as DP3‐C). Collectively, our results demonstrated that our computer modeling‐based screening method for evaluating the aggregation process and binding energy was successful. This method effectively distinguished the differences among peptides in our library. According to the results, DP2‐C, DP3‐C, DP4‐C, DP7‐C, and DP10‐C exhibit greater self‐assembly and aggregation than other peptides, so they might form micelles in water and serve as ideal siRNA delivery vectors. Based on the above evidence, we selected DP2‐C, DP3‐C, DP4‐C, DP7‐C, and DP10‐C to synthesize and confirm that the method we established is accurate and reliable.

### 2.2 Characterization of Candidate Peptides

**Figure 2:**
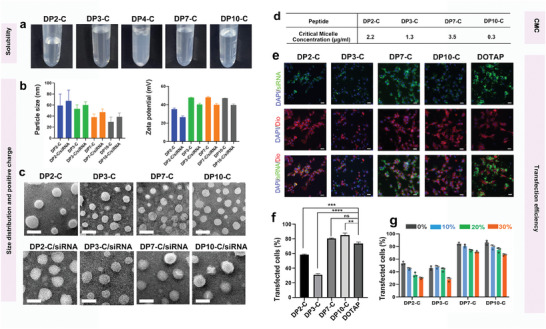
The characteristics of different cationic peptides in vitro. a) Incubation with PBS led to peptide aggregation and precipitation. b) Zeta potential distribution and size distribution of peptides and peptides/siRNA (*n* = 3, data are presented as means ± SDs). c) Morphology of each peptide and peptides/siRNA under TEM. Scale bars:100 nm. d) CMCs of peptides determined by fluorescence spectrophotometer with Nile Red. e,f) Fluorescence images (e) of 293 cells after FAM‐siNC transfection using different peptides and the efficiency of transfection analyzed by flow cytometry (f) (Red, Dio; Green, siRNA; Blue, DAPI; Scale bars: 200 µm) (*n* = 3, data are presented as means ± SDs, and the significant differences were analyzed by two‐tailed unpaired t‐test. ns, not significant; ^**^ *p* < 0.01; ^***^ *p* < 0.001; ^****^ *p*< 0.0001). g) Changes in the transfection efficiency of each peptide in the serum environment (*n* = 3, data are presented as means ± SDs).

To verify our computer‐aided prediction, the abovementioned DP2‐C, DP3‐C, DP4‐C, DP7‐C, and DP10‐C were synthesized. We first investigated the ability of these materials to form micelles in water. DP2‐C, DP3‐C, DP7‐C, and DP10‐C formed a clear solution at a concentration of 20 mg ml^−1^ in water at room temperature, while DP4‐C was almost insoluble in water (**Figure** **2**a). We further analyzed the dispersion of the soluble peptides after dissolution. Our results revealed that all the peptides had a particle size distribution within the range of 30–100 nm (Figure 2b), and a sphere‐like morphology was observed in all the soluble peptide samples via transmission electron microscopy (TEM) (Figure 2c). Additionally, DP3‐C, DP7‐C, and DP10‐C exhibited a uniform size distribution and morphology of approximately 50 nm, while DP2‐C exhibited an uneven size distribution with sporadic large particles. In addition, all of the above peptides were positively charged in water, ranging from 30 to 50 mV (Figure 2b). Therefore, our experiments suggested that all soluble peptides can form micelles and have a positive charge in water, which indicated that they have potential for siRNA delivery.

Subsequently, the siRNA delivery capacities of the four peptides were evaluated using gel shift assays. As shown in Figure S2a (Supporting Information), all the peptides completely blocked the siRNAs in a specific proportion. When the molar ratio of DP2‐C:siRNA reached 2:1 (w/w), the DP3‐C:siRNA ratio reached 2:1 (w/w), the DP7‐C:siRNA ratio reached 5:1 (w/w), and the DP10‐C:siRNA ratio reached 4:1 (w/w), almost no siRNA band was observed. These results indicated that our peptides bound siRNA effectively and efficiently, further reflecting their potential as siRNA delivery vectors. After the peptides were bound to siRNA, the particle sizes increased to approximate values of 67.86±19.62 nm (DP2‐C/siRNA), 60.32±5.86 nm (DP3‐C/siRNA), 47.21±5.98 nm (DP7‐C/siRNA) and 38.50±7.32 nm (DP2‐C/siRNA) (Figure 2b). Although the zeta potentials of the complexes were lower than that of the peptide alone, the surface charges were 26.45±1.47 mV (DP2‐C/siRNA), 40.29±1.49 mV (DP3‐C/siRNA), 39.99±1.41 mV (DP7‐C/siRNA) and 39.76±1.10 mV (DP10‐C/siRNA) (Figure 2b). Moreover, through TEM, we observed that all the peptide–siRNA mixtures formed micelles (Figure 2c). These observations suggested that the candidate peptides can form complexes with siRNA. Additionally, protection of siRNAs from enzymatic degradation by delivery vectors is critical for systemic administration. Thus, an RNase hydrolysis assay was performed. In contrast to those of the naked siRNA, the siRNAs extracted from all the peptide–siRNA complexes could be clearly observed on the gel, illustrating that each peptide protected the siRNA from RNase degradation to different degrees. Among these peptides, the siRNA within the DP7‐C complex was efficiently protected from enzymatic degradation for as long as 4 h, while DP2‐C and DP3‐C maintained protection for only 0.5 h, which suggested that the different peptides have different protective abilities due to their variable amino acid compositions (Figure S2b, Supporting Information). Therefore, we continued to verify the ability of these peptides to deliver siRNA. The critical micelle concentration (CMC), which is an important surface activity parameter, was determined using a fluorescence spectrophotometer with Nile Red as a probe. The results showed that the CMCs of each peptide were 2.2 µg ml^−1^ (DP2‐C), 1.3 µg ml^−1^ (DP3‐C), 3.5 µg ml^−1^ (DP7‐C) and 0.3 µg ml^−1^ (DP10‐C) (Figure 2d). As shown in Figure 2e, all the peptides had significantly different siRNA transfection efficiencies. DP2‐C and DP3‐C had moderate transfection capacities lower than 50%, while DP7‐C and DP10‐C had transfection efficiencies greater than 75% and greater than that of DOTAP. Moreover, as shown in Figure 2e, the siRNA fluorescence signal was clearly distributed inside the cell membrane after delivery by the DP7‐C and DP10‐C peptides, and compared to the other peptides, DP7‐C and DP10‐C demonstrated more efficient transmembrane uptake and stronger siRNA uptake signals. Flow cytometry also revealed that the DP7‐C and DP10‐C peptides had relatively high siRNA uptake (Figure 2f). Moreover, the presence of high concentrations of serum had little impact on the transfection efficiency of DP7‐C or DP10‐C. However, a marked reduction in the siRNA delivery efficiency of DP2‐C and DP3‐C was detected when the cells were exposed to serum (Figure 2g). Our results suggested that these peptide candidates identified by computer simulation filtering methods have satisfactory dispersion ability and micelle formation in water. All of these peptides bind and protect siRNA but have different siRNA delivery efficiencies. Thus, we successfully identified peptides with high siRNA delivery potential in our peptide library through a computer simulation screening method, indicating that our computer modeling‐based method predicted the properties of peptides with high accuracy.

### 2.3 Safety Evaluation of Peptides

**Figure 3:**
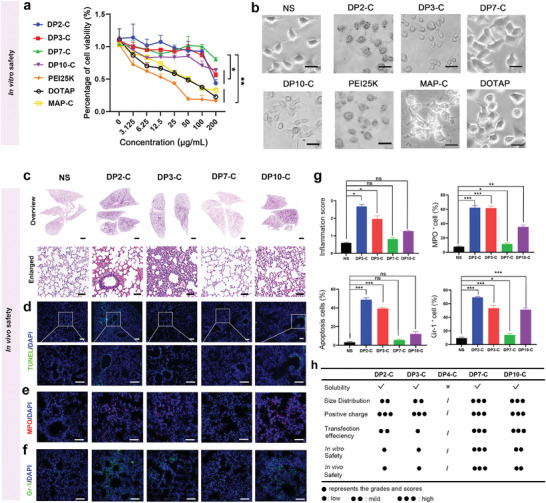
The cytotoxicity test of candidate peptides. a) Comparison of cytotoxicity of each nano‐vector by MTT assay (*n* = 3, data are presented as means ± SDs, and the significant differences were analyzed by one‐way ANOVA with Tukey's multiple comparisons test. ^*^ *p* < 0.05; ^**^ *p* < 0.01). b) Morphological changes in the cells treated with various vectors; Scale bars: 25 µm. c–f) C57BL/6 mice were injected with various cationic peptides (*n* = 3). Hematoxylin‐eosin (HE) staining (c), TUNEL staining (d), and immunofluorescence staining of MPO+ (e) and Gr‐1^+^ cells (f) in representative mouse lung sections 24 h after injection are presented. g) Histogram of (c–f). (*n* = 3, data are presented as means ± SDs, and the significant differences were analyzed by two‐tailed unpaired t‐test. ns, not significant, ^*^ *p* < 0.05, ^**^ *p* < 0.01, ^***^ *p* < 0.001, ^****^ *p*< 0.0001). h) Comparison of the characteristics of predicted peptides. The number of dots represents the score.

Since toxicity is a key factor of peptides as gene delivery vectors, we further evaluated the safety of candidate peptides in vitro and in vivo. We assessed the inhibition of cell proliferation by the peptides. Compared with PEI25K, our peptides showed obvious cell safety at 4 and 24 h (Figure S3, Supporting Information). With increasing peptide concentration and time (72 h), DP2‐C and DP3‐C strongly inhibited cell proliferation (**Figure** **3**a). Moreover, DP2‐C‐ and DP3‐C‐treated A549 cells exhibited swollen cytoplasm and ruptured plasma membranes at 72 h, suggesting a necrosis‐like morphological change (Figure 3b). These data showed that peptides selected from the library have varying degrees of in vitro safety.

To further compare in vivo toxicity, the lung tissues of mice after intravenous injection of peptides were analyzed. The results showed that different degrees of pulmonary inflammation, including massive inflammatory cell infiltration and pulmonary hyperemia, could be observed in mice injected with each of the peptides except DP7‐C; among these, DP2‐C and DP3‐C caused more severe inflammation, while the inflammation caused by DP10‐C was relatively moderate (Figure 3c). Moreover, the terminal deoxynucleotidyl transferase‐mediated deoxyuridine triphosphate (dUTP) digoxigenin nick‐end labeling (TUNEL) assay revealed that there were fewer apoptotic cells in mice treated with DP7‐C‐ (5.64%) or DP10‐C (12.08%) than in mice treated with DP2‐C (48.76%) or DP3‐C (39.35%) (Figure 3d). Since acute lung inflammation is believed to be associated with neutrophil infiltration, the infiltration of neutrophil‐like cells that stained positive for MPO^+^ and Gr‐1^+^ was characterized. Compared to those in the lung tissues from normal mice, markedly greater numbers of MPO^+^ and Gr‐1^+^ cells were observed in lung tissues from mice injected with DP2‐C (MPO^+^ 62.27%; Gr‐1^+^ 68.36%) or DP3‐C (MPO^+^ 61.71%; Gr‐1^+^ 53.25%) (Figure 3e,f). Similarly, according to the HE and TUNEL results, lung tissues from mice treated with DP7‐C (MPO^+^ 11.60%; Gr‐1^+^ 14.13%) or DP10‐C (MPO^+^ 35.35%; Gr‐1^+^ 39.05%) exhibited decreased neutrophil accumulation (Figure 3e,f). Therefore, the peptide candidates obtained by computer‐aided screening presented different degrees of in vivo safety (Figure 3g). Specifically, DP7‐C and DP10‐C performed particularly well and prevented obvious lung injury, such as lung cell apoptosis and neutrophil aggregation, which can lead to lung failure (Figure 3h). Overall, DP7‐C and DP10‐C further exhibited potential for application as siRNA delivery vectors in vivo.

### 2.4 Single‐Cell RNA‐Seq Analysis of Transcriptomic Profiles of Livers and Lungs Exposed to Cationic Vectors

DP7‐C and DP10‐C had similar effects on the lung inflammatory response and lung injury, and their in vivo toxicities were not significantly different. Accordingly, we attempted to distinguish their toxic effects at the molecular level. Single‐cell sequencing (scRNA‐seq) provides an unbiased view of the transcriptome and covers a broad dynamic range of differentially expressed genes at single‐cell resolution.^[^ ^27^ ^]^ Therefore, we used scRNA‐seq to analyze lung and liver samples from mice after vector injection of DP7‐C, DP10‐C, DOTAP and MAP‐C. DOTAP is a classic cationic siRNA vector, and MAP‐C has similar physicochemical properties to peptides in our library (data not shown); therefore, both were used as positive controls in our study.

**Figure 4:**
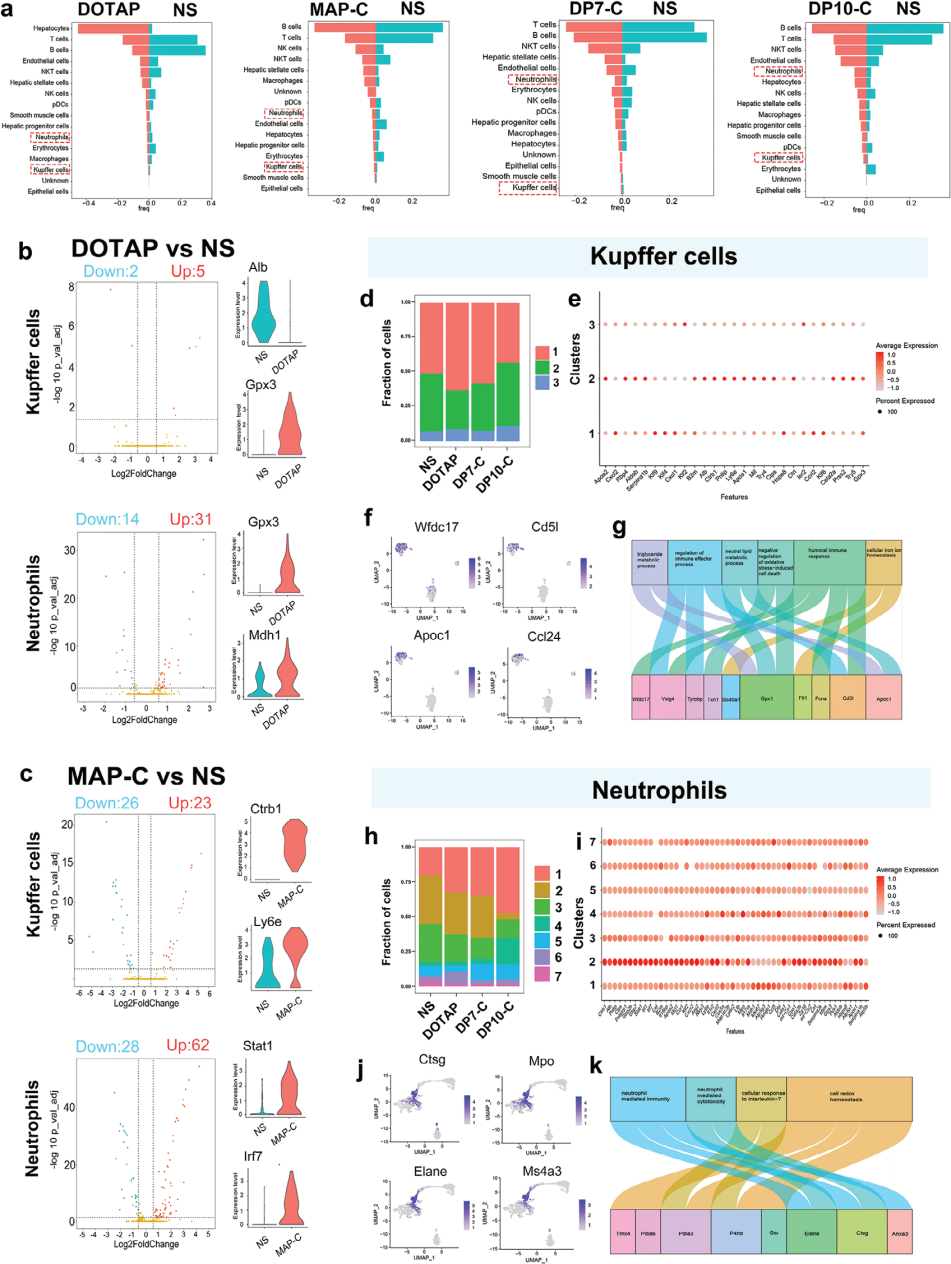
Distinct gene expression patterns and ratios of cells in the liver after systemic administration of peptides. a) Quantitative results of various cell types in liver cells (|logFC| ≥ 0.25 and adjusted *p* value ≤ 0.05). b,c) Volcano plot of differential gene expression between the NS and peptide‐administered groups. Violin plots of representative inflammation‐associated gene expression in Kupffer cells and neutrophils d) Fraction of cell types originating from Kupffer cells (|logFC| ≥ 0.25 and adjusted P value ≤ 0.05). e) Dot plot of differential gene expression in Kupffer cell subtype cells of NS, DP10‐C and DP7‐C. The color and size indicate the effect size. f) Highlighted t‐SNE plot of representative genes in Kupffer cells. g) Sankey diagram illustrating peptide administration‐induced changes in the expression of components of inflammation‐ and blood coagulation‐related signaling pathways in Kupffer cell subtypes. h) Fraction of cell types originating from neutrophils. (h) Fraction of cell types originating from neutrophils (|logFC| ≥ 0.25 and adjusted *p*‐value ≤ 0.05). i) Dot plot of differential gene expression in subtypes of neutrophils after NS, DP10‐C and DP7‐C administration. The color and size indicate the effect size. j) Highlighted t‐SNE plot of representative genes in neutrophils. k) Sankey diagram illustrating peptide administration‐induced changes in the expression of components of inflammation‐ and blood coagulation‐related signaling pathways in neutrophil subtypes.

First, cells were clustered based on their expression profile, and cell types were annotated based on established cell markers (Figure S4, Supporting Information). A total of 16 clusters were identified in the liver corresponding to five major cell groups: epithelial cells, endothelial cells, macrophages, hepatocytes, and neutrophils (Figure S5, Supporting Information). We observed dynamic changes in the cellular composition between the normal control (saline‐injected) and vector‐injected livers. Compared to the normal control group, the DOTAP‐treated group exhibited a sharp increase in the number of neutrophils and a decrease in the number of immune cells, such as T cells and B cells (**Figure** **4**a). MAP‐C primarily affects endothelial cells, erythrocytes and macrophages (including Kupffer cells, a specific kind of liver macrophage) (Figure 4a). DP7‐C has certain effects on immune cells, including T cells, B cells and NKT cells but has little effect on blood cells and inflammatory cells, such as neutrophils and macrophages (Figure 4a). DP10‐C and DP7‐C presented similar effects on immune cells, but DP10‐C resulted in a higher number of inflammatory cells and fewer erythrocytes decreased (Figure 4a). We explored the major changes that occurred in macrophages and neutrophils between the untreated group and vector‐injected group and further analyzed the differential gene expression in the two cell populations. In Kupffer cells, DOTAP and MAP‐C affected 7 and 49 genes, respectively, downregulating *Apoa2* and *Alb* and upregulating *Ly6e* and *Mif*, which indicated that inflammation was activated but that metabolic processes were inhibited by DOTAP and MAP‐C treatment (Figure 4b,c). In addition, compared to the liver tissue from the mice in the untreated group, the liver tissue from the mice in the DP10‐C group had 25 differentially expressed genes (DEGs) (Figure S6a, Supporting Information). Interestingly, the inflammatory genes *Cxcl2*, *Serpina1b*, and *Klf2* and the metabolic process genes *Apoa2* and *Aldob* were downregulated, suggesting that DP10‐C impaired inflammation and metabolism (Figure S6a, Supporting Information). Notably, DP7‐C hardly caused any decrease in gene expression in Kupffer cells, and only 7 genes were upregulated, suggesting that DP7‐C minimally affects Kupffer cells (Figure S6a, Supporting Information). In contrast, for neutrophils, compared with the untreated group, the DP10‐C treatment group exhibited 542 DEGs (Figure S6a, Supporting Information). In contrast to the DEGs in Kupffer cells, the inflammatory genes *Mpo*, *Ly6c2*, and *Ly6e* were upregulated, suggesting that DP10‐C triggers the inflammatory reaction (Figure S6a, Supporting Information). The DP7‐C group also exhibited 27 DEGs, such as *mt‐Co1* and *mt‐Co2*, which are involved in electron‐coupled proton transport. DOTAP (45 DEGs) and MAP‐C (90 DEGs) still induced inflammation and impaired lipid metabolism (Figure S6a, Supporting Information). Moreover, signaling pathway analysis demonstrated that the DEGs in the DOTAP and MAP‐C treatment groups were involved in metabolism and lipid transport pathways and that DP10‐C disrupted erythrocyte development and homeostasis and triggered inflammatory reactions (Figure S7, Supporting Information). Interestingly, DP7‐C affects the rRNA process, which is unrelated to cytotoxicity (Figure S6b, Supporting Information). Thus, we verified that our designed vectors DP10‐C and DP7‐C have greater biosafety than do the classic cationic MAP‐C and DOTAP vectors.

Considering that changes in the number of neutrophils and Kupffer cells were more obvious in the different treatment groups and that numerous studies have demonstrated their function in nanoparticle‐induced tissue injury,^[^ ^28^, ^29^, ^30^, ^31^, ^32^ ^]^ we focused on changes in the DP10‐C and DP7‐C treatment groups. We identified distinct cell subtypes by recruiting neutrophils and Kupffer cells separately. Kupffer cells were divided into three subtypes based on the characteristic genes, and the resulting gene expression heatmap represented the top marker genes for each of those clusters (Figure 4d,h). Compared to the untreated group, the DOTAP and DP10‐C groups exhibited sharply increased numbers of *Apoc1* ^+^ and *Ms4a1* ^+^ Kupffer cells, but the two cell types in the DP7‐C group only slightly changed (Figure 4d–f; Figure S6c, Supporting Information). GO enrichment analysis revealed that the DEGs in the DP10‐C subgroup were associated with Kupffer cell migration, activation and differentiation, collagen deposition and blood vessel diameter regulation (Figure 4g; Figure S6d, Supporting Information). Additionally, neutrophils were classified into seven subtypes (Figure 4h,i), and the numbers of *Irf8* ^+^, *Il1β* ^+^, *Ccl12* ^+^ and *Ctsg* ^+^ neutrophils significantly increased, while the number of *Il6* ^+^ neutrophils decreased markedly in the DOTAP and DP10‐C groups (Figure 4h–j; Figure S6e, Supporting Information). GO analysis revealed that the DEGs between the DP7‐C and DP10‐C groups were enriched in pathways related to artery morphogenesis, cytokine production and cell apoptosis (Figure 4; Figure S6f, Supporting Information). The DP7‐C subgroup exhibited only a subtle change in the ratio of cell subtypes and no significant gene and pathway variation compared to those in the untreated group (Figure 4). These results suggested that DP10‐C activated the inflammatory response and disrupted lipid metabolism in the liver and that DP7‐C resulted in less liver injury.

**Figure 5:**
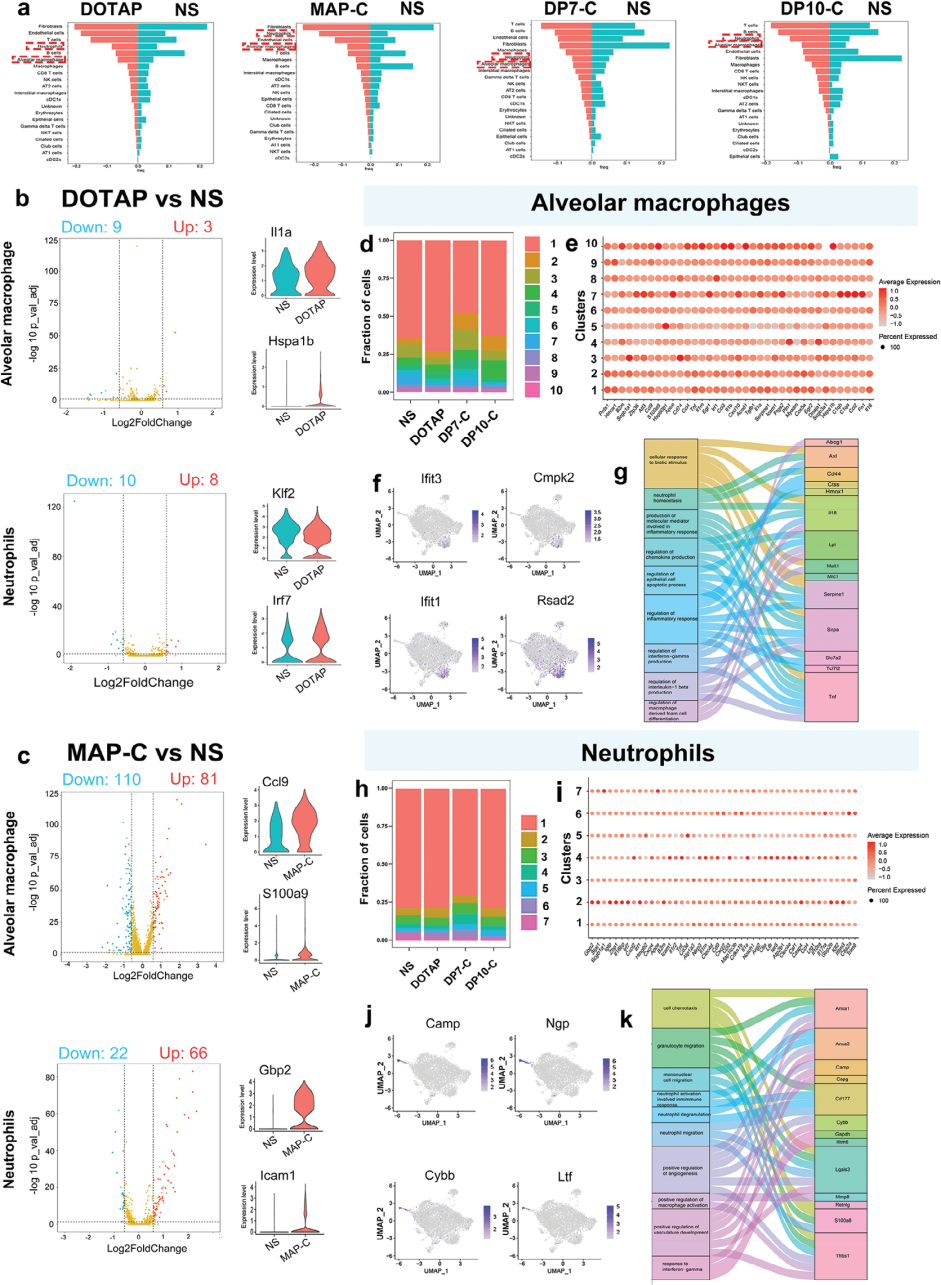
Systemically administered peptides altered cell function in the lung. a) Quantitative analysis of various cell types in the lung (|logFC| ≥ 0.25 and adjusted *p*‐value ≤ 0.05). b,c) Violin diagram of the expression of genes related to alveolar macrophages and neutrophils in the lung in the NS and peptide‐treated groups (|logFC| ≥ 0.25 and adjusted *p*‐value ≤ 0.05, data are presented as means ± SDs). d) Subgrouping analysis of alveolar macrophages in the lungs of NS‐, DP10‐C‐ and DP7‐C‐treated mice. e) Dot plot of representative gene expression in each cluster in alveolar macrophages. The color and size indicate the effect size. f) The expression highlights of representative genes in alveolar macrophages. g) Selected gene sets that were significantly enriched in RNA‐seq expression profiles associated with alveolar macrophages of DP7‐C‐treated lungs compared to DP10‐C‐treated lungs. h) Subgrouping analysis of neutrophils in the lungs of NS‐, DP10‐C‐ and DP7‐C‐treated mice (|logFC| ≥ 0.25 and adjusted *p*‐value ≤ 0.05). i) Dot plot of representative gene expression in each neutrophil cluster. The color and size indicate the effect size. j) The expression highlights of representative genes in neutrophils. k) Selected gene sets that were significantly enriched in RNA‐seq expression profiles associated with neutrophils of DP7‐C‐treated lungs compared to DP10‐C‐treated lungs.

From the lung tissue transcriptomic data obtained from scRNA‐seq, 21 clusters were classified (Figure S8, Supporting Information). Similarly, we analyzed the cell cluster distribution in all the groups. Similarly, compared with those in the untreated group, the DOTAP and MAP‐C groups exhibited a sharp increase in the number of neutrophils and alveolar macrophages and a decrease in the number of immune cells and endothelial cells (**Figure** **5**a; Figure S9, Supporting Information). DP7‐C had certain effects on fibroblasts and epithelial cells but had little effect on immune cells, blood cells or inflammatory cells, such as neutrophils and macrophages (Figure 5a). DP10‐C had similar effects on immune cells and fibroblasts to those of DP7‐C but had a greater effect on inflammatory cells and a decreased effect on erythrocytes (Figure 5a). We identified the major changes that occurred in alveolar macrophages and neutrophils between the untreated group and vector‐injected groups; thus, we further analyzed the differential gene expression in the two cell populations. In alveolar macrophages, DOTAP and MAP‐C affected the expression of 12 and 191 genes, respectively, including the upregulation of *Il1a*, *Hspa1b*, *Ccl9*, and *S100a9*, indicating that inflammation was activated by DOTAP and MAP‐C (Figure 5b,c). In addition, compared with those from untreated mice, lung tissue from mice treated with DP10‐C had 126 DEGs (Figure S10a, Supporting Information). Interestingly, the inflammatory genes *Tnf* and *Cxcl10* were both upregulated, suggesting that DP10‐C induced lung inflammation (Figure S10a, Supporting Information). Notably, although DP7‐C downregulated 36 genes and upregulated 20 genes in alveolar macrophages, these genes are pseudogenes associated with 18S RNA and have no biological function in the lung, suggesting that DP7‐C has minimal impact on alveolar macrophages (Figure S10a, Supporting Information). In contrast, for neutrophils, DP10‐C treatment resulted in the differential expression of 359 genes (Figure S10a, Supporting Information). In contrast to the DEGs in alveolar macrophages, the inflammatory genes *Ccl3* and *Nlrp3* were upregulated, suggesting that DP10‐C triggers an inflammatory reaction (Figure S10a, Supporting Information). DP7‐C treatment also slightly affected gene expression, resulting in 27 DEGs, and the inflammatory gene *Casp4* was downregulated. DOTAP (18 DEGs) and MAP‐C (88 DEGs) still induced inflammation and impaired lipid metabolism (Figure 5b). Moreover, signaling pathway analysis demonstrated that the DEGs between the DOTAP and MAP‐C groups were involved in macrophage differentiation, the inflammatory response and blood coagulation (Figure S11, Supporting Information). The DEGs in the DP10‐C subgroup were involved in neutrophil migration and the acute‐phase response (Figure S10b, Supporting Information). Interestingly, DP7‐C affects the temperature stimulus and protein processing, which are unrelated to cytotoxicity (Figure S10b, Supporting Information). Thus, we verified that our designed vectors DP10‐C and DP7‐C have greater safety than MAP‐C and DOTAP.

There were significant differences in the numbers of alveolar macrophages and neutrophils in all the treated groups, and we constructed volcano plots of the DEGs from peptide‐treated mice to show that many of the upregulated genes in the two cell types were inflammation‐related genes. We further focused on subtle changes in alveolar macrophages and neutrophils. The macrophages were divided into 10 specific cell types based on the expression of the characteristic genes. Notably, 73% of the DOTAP‐treated alveolar macrophages and 62% of the DP10‐C‐treated alveolar macrophages were distributed in the *Sirpa* ^+^ cluster, whereas only 46% of the DP7‐C‐treated and 47% of the untreated alveolar macrophages were distributed in the *Sirpa* ^+^ cluster (Figure 5d–f). In contrast, the majority of untreated and DP7‐C‐treated alveolar macrophages were distributed within the *Cxcr6* ^+^ and *Cd79a* ^+^ clusters (Figure 5d–f; Figure S10c, Supporting Information). This phenotype correlated with cytokine receptor binding, vascular development and the NF‐κB pathway (Figure 5g; Figure S10d, Supporting Information). Neutrophils were grouped into 7 subclusters; among them, the numbers of *Ifit3* ^+^ and Camp^+^ neutrophils noticeably increased, and the number of *Bcl2a1a* ^+^ neutrophils decreased; moreover, in the DP10‐C group, the numbers of *Irf8* ^+^, *Il1β* ^+^, *Ccl12* ^+^ and *Ctsg* ^+^ neutrophils significantly increased, while the number of *Il6* ^+^ neutrophils decreased markedly (Figure 5h–j; Figure S10e, Supporting Information). GO analysis revealed that the DEGs between the DP7‐C and DP10‐C groups were enriched in pathways associated with granulocyte migration and neutrophil degranulation (Figure 5k; Figure S10f, Supporting Information). The DP7‐C group exhibited only a subtle change in the ratio of cell subtypes and insignificant gene and pathway differences compared to those of the untreated group (Figure 5h,i). Furthermore, the proinflammatory factors *Il1a*, *Tnf* and *Ccl4* were more enriched in the *Bcl2a1a* ^+^ cells in the DP10‐C‐treated group. These results suggested that DP10‐C treatment promotes the activation of the inflammatory response and coagulation process and contributes to the development of vascular disorders in the lungs, while DP7‐C treatment results in less lung injury.

In summary, we used scRNA‐seq to obtain extensive information about nanoparticle‐induced intracellular signaling and response pathways at the molecular level. All the vectors affected the studied tissues, but the different vectors affected different cell groups, which may be related to the composition of each peptide. Furthermore, we demonstrated in previous studies that macrophages and neutrophils are the main effector cell populations that promote inflammation and induce apoptosis and are major targets of gene vectors in tissues. In addition, we identified novel mechanisms, such as platelet aggregation and blood vessel morphology, associated with nanotoxicity. More importantly, for the first time, we used scRNA‐seq technology to investigate the liver and lung transcriptomic profiles following systemic administration of different vectors. With a single transcriptomic dataset, we could simultaneously analyze the expression of thousands of transcripts, which has much more analytic power than the detection of markers via conventional assays. Our study suggested that systemically administered MAP‐C and DOTAP can act as exogenous stimuli to recruit and activate inflammatory cells, including macrophages and neutrophils, and impair epithelial cell function. Moreover, specific differences between DP10‐C and DP7‐C were identified in terms of both cell numbers and DEGs, which confirmed that DP7‐C is a safe cationic vector for intravenous injection.

### 2.5 Systemic Delivery of Multiple DP7‐C/siRNA Complexes for Cancer Treatment

**Figure 6:**
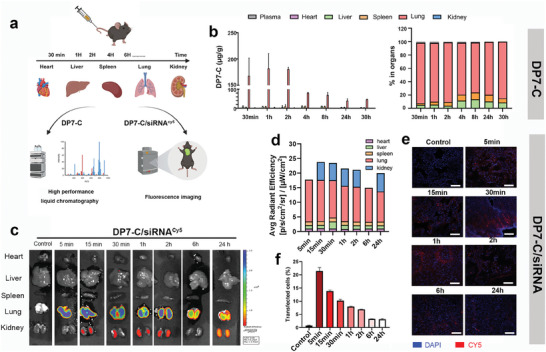
In vivo distribution of DP7‐C and DP7‐C/siRNA complex. a) Schematic illustration of the selective targeting of DP7‐C to lung tissue. b) The concentration of DP7‐C in major organs at different time points after intravenous injection was measured via mass spectrometry (*n* = 3, data are presented as means ± SDs). c) Fluorescence imaging results of the main organs from mice that were intravenously injected via the tail with DP7‐C/cy5‐siRNA solution at different time points (*n* = 3). d) Statistical result of (c). e) Frozen sections and flow cytometry analysis of the lung tissues from (c), (*n* = 3). (Red, Cy5; Blue, DAPI; Scale bars: 20 µm). f) Flow cytometric analysis of the proportion of Cy5+ cells in the lung (*n* = 3, data are presented as means ± SDs).

Based on the above results, DP7‐C was selected as a candidate for systemic siRNA delivery. To define the possible therapeutic target in vivo, we first characterized the tissue distribution of DP7‐C and the DP7‐C/siRNA complex after intravenous injection (**Figure** **6**a). We used liquid chromatography tandem high‐resolution mass spectrometry (LC‐HRAM) to analyze the specific distribution and concentration of DP7‐C in the main organs. According to our results, DP7‐C was specifically enriched in lung tissue, and its accumulation was maintained until 24 h after intravenous injection (Figure 6b). Additionally, the extent of DP7‐C distribution in lung tissue was significantly greater than that in other tissues, and the proportion was greater than 85% (Figure 6b). In summary, DP7‐C could be specifically distributed in lung tissue after intravenous injection, suggesting the possibility of a lung‐targeted delivery system.

We subsequently verified whether the DP7‐C/siRNA complex had a similar distribution. A similar study was performed, but this experiment included the intravenous injection of the DP7‐C/siRNA^cy5^ complex. Shortly after administration, intense fluorescent signals were detected in the lung tissues, and the peak fluorescence signal was maintained at 1.4×10^8^ within 1 h (Figure 6c,d). The DP7‐C/siRNA^cy5^ complex fluorescence signal in the lung accounted for more than 72% of the total signal (Figure 6c,d). Frozen sections of lung tissue collected at different times postinjection exhibited strong and durable fluorescent signals (Figure 6e). Moreover, flow cytometry results also demonstrated lung targeting and long retention of siRNA in the lung (Figure 6f).

**Figure 7:**
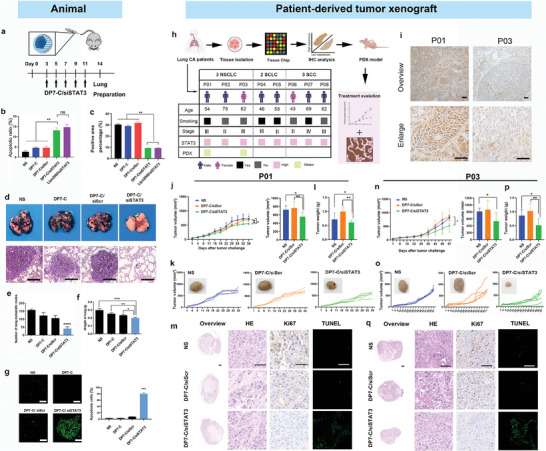
The therapeutic effect of the DP7‐C/siSTAT3 complex on lung cancer models. a) siRNAs targeting mouse STAT3 were selected and complexed with DP7‐C. b) Antiproliferative effect of DP7‐C/siSTAT3 determined by a clonogenic assay. The number of clones was calculated for each well (*n* = 3, data are presented as means ± SDs, and the significant differences were analyzed by one‐way ANOVA with Tukey's multiple comparisons test. ns, not significant; ^**^ *p* < 0.01). c) Apoptosis induction ability of DP7‐C/siSTAT3 determined by flow cytometry. (*n* = 3, data are presented as means ± SDs, and the significant differences were analyzed by one‐way ANOVA with Tukey's multiple comparisons test. ^**^ *p* < 0.01). d–g) Therapeutic effect of DP7‐ C/siSTAT3 complex. Parameters including lung tissue pictures (d), number of metastasis nodules (e), lung weight (f) and degree of apoptosis (g), were evaluated. Scale bars: 20 µm (*n* = 5, data are presented as means ± SDs, and the significant differences were analyzed by two‐tailed unpaired *t*‐test. ^*^ *p* < 0.05; ^**^ *p* < 0.01; ^***^ *p* < 0.001; ^****^ *p*< 0.0001). h) Schematic diagram highlighting the workflow of the study design and analysis. i) Immunohistochemical staining results of tumor tissues from patients. Scale bars: 100 µm. j–m) Therapeutic effect of the DP7‐C/siSTAT3 complex in P01. Tumor growth curves of each group (j,k); average tumor weights at the end of treatment (l); immunohistochemical staining detection of tumor tissues from all groups (m). (*n* = 5, data are presented as means ± SDs, and the significant differences were analyzed by one‐way ANOVA with Tukey's multiple comparisons test. ^*^ *p* < 0.05; ^**^ *p* < 0.01). n–q) Therapeutic effect of the DP7‐C/siSTAT3 complex in P03. Tumor growth curves of each group (n,o); average tumor weights at the end of treatment (p); immunohistochemical staining detection of tumor tissues from all groups (q). (*n* = 5, data are presented as means ± SDs, and the significant differences were analyzed by one‐way ANOVA with Tukey's multiple comparisons test. ^*^ *p* < 0.05; ^**^ *p* < 0.01).

To further evaluate the therapeutic potential of the DP7‐C vector upon systemic delivery, siRNAs targeting mouse STAT3 were selected and complexed with DP7‐C micelles (**Figure** **7**). DP7‐C‐based systemic delivery was attempted with siRNA targeting STAT3, which has been demonstrated to be a key transduction and transcription factor in cancer progression.^[^ ^33^, ^34^ ^]^ The anticancer effects of these compounds were characterized using a B16F10 cells and mouse lung metastasis melanoma mode (Figure 7a). Lipo2000, a commercialized vector for gene delivery, was used as the positive control in vitro. Compared with Lipo2000/siSTAT3, DP7‐C/siSTAT3 also achieved favorable in vitro antitumor effects, including high efficacy in inhibiting tumor cell proliferation and inducing tumor cell apoptosis (Figure 7b,c). Moreover, in a mouse lung cancer model, the group that received systemically administered DP7‐C/siSTAT3 exhibited better therapeutic results than the untreated group (NS), nanoparticle control group (DP7‐C) and scramble group (DP7‐C/siScr) (Figure 7c). Obvious cancer suppression was also observed when the DP7‐C/siSTAT3 complex was systemically administered (Figure 7d). The DP7‐C/siSTAT3 complex had therapeutic effects on both the lung weight and the number of B16F10 tumor nodules (Figure 7e,f). A strong apoptosis‐inducing effect was revealed by TUNEL staining of lung tissue sections (Figure 7g). Furthermore, no significant impairment of liver or kidney function was observed after intravenous treatment with DP7‐C or the DP7‐C/siRNA complex (Figure S12, Supporting Information). Overall, these results demonstrated that systemic administration of DP7‐C micelles facilitated the accumulation of the delivered siRNA in lung tissue and thus effectively promoted therapeutic effects in a mouse model of lung cancer.

### 2.6 Therapeutic Effect of the DP7‐C/siSTAT3 Complex in Population‐Level Patient‐Derived Tumor Xenograft (PDX) Models

PDX models are widely used as a drug discovery platform with high predictive clinical efficacy.^[^ ^35^ ^]^ To further investigate the effect of DP7‐C as a siRNA delivery vector, we used clinical samples derived from 8 patients with lung cancer (including adenocarcinoma and squamous carcinoma) to construct lung cancer xenograft models (Figure 7h). In this experiment, we intratumorally injected siSTAT3 loaded by DP7‐C in situ into mice twice a week. First, we performed IHC to distinguish whether STAT3 was expressed and established STAT3‐expressing models (Figure 7i). In the P01 model, DP7‐C/siSTAT3 showed potent and significant tumor inhibitory effects, with the slowest tumor growth rate (Figure 7j,k). After treatment, an obvious decrease in tumor volume was observed in the DP7‐C/siSTAT3 group (558.74±107.65 mm^3^) compared to the NS group (723.52±96.99 mm^3^, *p* = 0.0159) and DP7‐C/siScramble group (767.89±152.70 mm^3^, *p* = 0.0079) (Figure 7l). The tumor inhibition rate reached 22.77% and 27.23% in the DP7‐C/siSTAT3 group compared with the NS group and DP7‐C/siScramble group, respectively. More importantly, the tumor weight of the DP7‐C/siSTAT3 group (0.58±0.04 g) was lighter than that of the NS group (0.73±0.17 g, *p* = 0.055) and DP7‐C/siScramble group (0.94±0.15 g, *p* = 0.007) (Figure 7l). IHC staining of Ki67 proved that DP7‐C/siSTAT3 treatment inhibited tumor cell proliferation, and TUNEL staining verified apoptotic function, revealing the underlying mechanism of the therapeutic effect (Figure 7m). Moreover, there was no obvious body weight loss or unforeseen events in the mice that were administered DP7‐C/siSTAT3 in all xenograft models tested in this study (Figure S13, Supporting Information).

In the P07 model, the DP7‐C/siSTAT3 treatment had a favorable antitumor effect on the dissected tumor (Figure 7n). A beneficial therapeutic outcome was observed in the DP7‐C/siSTAT3 group (533.08±246.71 mm^3^, *p* = 0.0159), and the average tumor volume in the DP7‐C/siSTAT3 complex‐treated group was significantly lower than that in the other groups, in which tumor growth was inhibited by >35% (Figure 7n,o). The tumor weights are shown in Figure 7i. The tumor weight of the DP7‐C/siSTAT3 group was 0.51±0.24 g, while that of the NS group was 0.86±0.21 g (*p* = 0.031), and that of the DP7‐C/siScramble group was 1.03±0.27 g (*p* = 0.016), indicating a satisfactory treatment effect (Figure 7p). IHC staining of Ki67 proved that DP7‐C/siSTAT3 treatment had antiproliferative and tumor cell apoptosis‐promoting effects (Figure 7q). In addition, body weight changes were not observed, and the major tissues exhibited normal architecture and cellularity in all groups, indicating that DP7‐C/siRNA treatment had negligible cytotoxic effects (Figure S13, Supporting Information).

## 3 Discussion

siRNA administration has shown tremendous potential for cancer immunotherapy, and various types of formulated nanomaterials have been explored for their ability to increase siRNA payloads and improve siRNA effects. One study^[^ ^36^ ^]^ constructed a caliprotein particle (CPP) with nine arginines (9R) and a macrophage‐targeted sequence that specifically binds to the neuropilin‐1 receptor on the macrophage surface, which achieved macrophage‐targeted RNA delivery. Another study^[^ ^37^ ^]^ reported that the incorporation of mannose into lipid nanoparticles (LNPs) with high PEG‐lipid content allowed selective delivery of mRNA/siRNA only to liver sinusoidal endothelial cells through the mannose‐CD206 interaction. These delivery vectors successfully achieved targeted cellular siRNA uptake. However, these vectors were directly synthesized with no prior evaluation; thus, not all vectors are ideal. Considering that a single peptide design is not cost‐effective and has a risk of failure, we established a peptide library containing 10 peptides, and natural amino acids were randomly combined in the peptide sequence. Since all peptide syntheses waste large amounts of reagents and impose an environmental burden, a computer‐based process was used to analyze the sequences in the entire peptide library to identify suitable candidates. This computer‐based method revealed a spectrum of basic peptide characteristics, consisting of nanostructure, hydrophobicity, conformational changes, and aggregation propensities. After computer modeling‐based high‐throughput screening, half of the library peptides were excluded. The five optimal peptides were synthesized with strategic sequences, and their solubility in water and nanocharacteristics were in substantial agreement with the computer predictions, demonstrating that our computer modeling approach is reliable. Further experiments were applied to evaluate the cellular uptake efficiency and cytotoxicity of the peptides. Surprisingly, DP7‐C performed markedly better than the other peptides and had a greater transfection rate and lower cytotoxicity. DP7‐C also presented excellent application prospects in RNAi therapy for lung cancer. The histidine residue provides endosomal escape, the arginine and lysine residues provide binding sites for nucleic acids, and hydrophobic amino acids can interact with hydrophobic parts of the cell membrane.^[^ ^38^ ^]^ Noticeably, the charge of the amino acid sequence is associated with transfection efficiency; however, cations can also be toxic to body cells by attaching to cell membranes and disrupting their functions.^[^ ^38^ ^]^ Thus, balancing the transfection rate and biosafety is still a challenge in peptide vector design. Our study used a peptide library to expand the search for available candidates and to investigate different amino acid combinations. After the computer‐based platform was used to construct self‐assembled nanoparticles, we investigated their nanotoxicity in vivo and in vitro by functional experiments. Cell viability in vitro, cell apoptosis and the inflammatory response in lung tissues after i.v. peptide administration were assessed to identify favorable candidates. Therefore, this study demonstrated a highly efficient and robust approach for screening top‐performance candidates from an entire peptide library, overcoming the limitations of a single design, candidate and evaluation method.

Nanoparticle toxicity following systemic administration has been a barrier to clinical application, and knowledge of the toxicological mechanism of these agents is extremely limited. To date, some research has focused on certain cells or specific pathological pathways and revealed comparable partial mechanisms. For example, Liu et al. reported that Ly6C^+^ inflammatory monocytes and increased PGE2 production induced by mtDNA via the TLR9‐ or STING‐mediated MAPK‐NF‐κB‐COX2 pathway may contribute to the regulation of the nanoparticle‐induced inflammatory response.^[^ ^39^ ^]^ Zhu et al. revealed that upconversion nanoparticles (UCNs), as model‐engineered nanomaterials, play a critical role in the nanotoxicity of Kupffer cells.^[^ ^26^ ^]^ Zhang et al. investigated whether SiO2 nanoparticles (NPs) induce retinal cell apoptosis, and glial cell activation, inflammation and ROS accumulation were evaluated as potential mechanisms of SiO2 NP‐induced retinal toxicity.^[^ ^40^ ^]^ Although these studies evaluated the biocompatibility and basic toxicological mechanism of the vector, their methods were confined to conventional assays, such as HE staining and cytotoxic marker detection. These technologies are broad and insufficient for elucidating the complexity and heterogeneity of cell–nano interactions. We exploited scRNA‐seq to comprehensively explore individual cells in response to CPP exposure. Moreover, the aggregation of nanoparticles in the lungs and liver can induce severe clotting, resulting in acute, fatal toxicity in mice,^[^ ^41^, ^42^ ^]^ and DP7‐C is distributed throughout the lung and liver. For the first time, we used scRNA‐seq to investigate the microenvironment in liver and lung tissues following systemic CPP administration. scRNA‐seq is widely used for analyzing the interactions between nanomaterials and cells to reveal changes in the proportions of various cell types and for high‐resolution profiling of differential cytokine expression and signaling responses after the administration of nanomaterials, with low interference from NPs.^[^ ^43^ ^]^ We analyzed the effects of CPPs on various cell populations, especially macrophages and neutrophils, not only in terms of number but also in terms of detailed pathways. Several previous studies have shown that scRNA‐seq is helpful for obtaining in‐depth knowledge on the interactions between biological tissues and nanoparticles.^[^ ^44^ ^]^ Regrettably, these studies are insufficient for elucidating the complexity and heterogeneity of cell–nano interactions. More importantly, the toxicity of DP7‐C and DP10‐C was not as obvious according to pathological examination of lung sections. Through scRNA‐seq, the following intracellular signaling and response pathways were revealed at the molecular level: DP10‐C regulated hemopoiesis and the neutrophil migration pathway, resulting in an increase in *Ccl12* ^+^ Kupffer cells, and increased interleukin family production, which was governed by *Sirpa* ^+^ alveolar macrophages. These results suggest that distinct cell subsets have different levels of cellular response and are involved in particular pathways that need to be considered during nanomaterial design. Furthermore, our innovative application of scRNA‐seq solved the limitations of previous reports; for instance, we explored NP toxicity mechanisms based on existing evidence or reasonable speculation, focused on NP‐induced cell apoptosis and acute inflammation, and described the phenomenon observed point by point. We focused on damaging effects, including blood coagulation and liver metabolic abnormalities, as well as subtle differences at the cellular level. Specifically, *Adrb2* is involved in cell adhesion and migration processes, including blood coagulation, host defense, and metastasis and is activated by i.v. DOTAP administration. Cationic peptides have been reported to cause serious side effects once in the bloodstream, and our results agree with previous conclusions, revealing that *Adrb2* may play an important role in hypercoagulability and pulmonary embolism. Moreover, we identified novel mechanisms associated with NP toxicity. The GTPase regulatory pathway is one of the most highly activated pathways. The GTPase superfamily of proteins provides molecular switches to regulate cell differentiation, vesicle trafficking and blood pressure. However, no study has mentioned the relationship between GTPase regulators and nanotoxicity. We discovered that the GTPase regulatory pathway was shared by the two cell populations, indicating that cationic compound exposure similarly disrupted blood vessel stabilization in both macrophages and neutrophils. Our study demonstrated that the use of scRNA‐seq in multiple organs is helpful for obtaining in‐depth knowledge of the interactions between immune cells and NPs and provides a novel risk assessment approach for future toxicity assessments of nanomaterials.

## 4 Experimental Section

### Structure Preparations and Molecular Dynamics Simulations

Cholesterol‐peptide molecules were prepared based on their sequences. Among the structures, the cholesterol structure was first prepared by Chemdraw16.0 with the substitution of 4‐(methylamino)−4‐oxobutanoate at the C3 position and then optimized under B3LYP/6‐31G* by the Gaussian09 program.^[^ ^45^ ^]^ Afterward, the HF/6‐31G* method and basis set were performed to evaluate the electrostatic potential, and then the obtained number was used to work out the restricted ESP of the molecules.^[^ ^46^ ^]^ The geometric characteristics of the substituted cholesterol were marked by the general amber force field (gaff).^[^ ^47^ ^]^ All of the peptides were described using the amber fb15 forcefield, and the solvent cholesterol‐peptide conjugates were analyzed by the fb3 TIP3P model. The cholesterol‐peptide molecules were constructed by the tleap program in Amber16. To randomly put multiple molecules into a system, Packmol software was applied. For one system, in total, 16 cholesterol‐peptide molecules (for all peptide systems) were randomly put into a 110×110×110 Å^−3^ box, and then 22 000 water molecules were put into the same box to fill the empty space. All systems were finally set up by the tleap program, and the molecular dynamics simulations were operated through the openmm MD package. The simulations included numerous steps. First was the minimization of the whole system, and then heat to 300 K by Langevin thermostat. The constant‐pressure ensemble (NPT) was performed in order to balance the system to 1 atmosphere pressure. Finally, extended MD simulations (100 ns) were run under the NPT.

### Preparation and Characterization of Peptide‐Cholesterol Conjugates

Peptide‐cholesterol conjugates were synthesized by modifying peptides with cholesterol at a 1:1 molar ratio at the N‐terminus. To explore the particle size and zeta potential of peptide micelles and peptide/siRNA, the Malvern ZetaSizer Nano‐ZS Zen3600 (Malvern Instruments, UK) was applied and three independent tests were performed. The morphologies of the prepared conjugates and related siRNA complexes were observed using TEM (JEOL JEM‐100CX, Japan).

### Evaluation of siRNA Transfection Efficiency

293 T cells (1×10^5^ cells per well) were cultured in a 24‐well plate. Then, we incubated DP7‐C, PEI25K and Lipo2000 with siRNA‐FAM (0.5 µg) respectively. After 10 min in room temperature (RT), the complexes were added to the cells carefully. After 4 h, the medium in the plates was replaced with different concentrations of FBS (including 0, 10%, 20%, and 30%). Twenty‐four hours later, each well was observed by light microscope, and the cell uptake efficiency was detected through flow cytometry.

### In Vivo and In Vitro Toxicity Analysis

For the evaluation of in vitro cell cytotoxicity, A549 cells were seeded into 96‐well plates (1×10^3^ cells per well) before being exposed to a series of concentrations of different cationic nanovectors. After 4 h, 24 h and 72 h, cell proliferation was assessed by MTT. In brief, 20 µL of MTT reagent was added and then incubated at 37°C. After 4 h, the MTT medium was disposed and 100 µL DMSO added. The absorbance was measured at 570 nm using the SpectraMax M5 Microtiter Plate Luminometer (Molecular Devices, USA).

For the evaluation of in vivo cell cytotoxicity, formalin‐fixed paraffin‐embedded lung tissues were prepared. Tissue sections were conducted with hydrogen peroxide (3%) at RT, and antigen restore was carried out by incubating sections with 20 mM sodium citrate at 95°C. The slides were immersed with primary antibodies against Gr‐1 (BD Biosciences, 1:50), MPO (Proteintech, 1:50), KI67 (Abcam, 1:50), F4/80 (CST, 1:50), and CD206 (CST, 1:50) at 4°C pass the night and then incubated 1 h with secondary antibodies at RT. Nuclei were stained with DAPI (Sigma–Aldrich). Immunofluorescence images were acquired using a Zeiss Bio fluorescence microscope. For immunohistochemistry, Primary antibodies were used, including anti‐STAT3 (CST, 1:100) and anti‐Ki67 (Abcam, 1:100).

### In Vivo Distribution of DP7‐C

For biodistribution studies, peptide (60 µg) alone or complexed with Cy5‐labeled siRNA (12 µg) was intravenously injected into C57BL/6 mice. At the indicated timepoint after intravenous injection, organs containing the heart, liver, spleen, lung, and kidney were isolated from administrated mice. Fluorescence signals were monitored and imaged using an IVIS Spectrum system (Caliper, Hopkinton, USA). Moreover, frozen sections of lung tissues were prepared for flow cytometry after fixation in 4% paraformaldehyde.

### Single‐Cell RNA Sequencing

Freshly tissues were washed in Hanks’ balanced salt solution, and cut into small pieces on ice, then digested with collagenase I and IV. After incubated for 30 min at 37°C with continuous shaking. The shredded tissues were filtered the 70 µm nylon mesh, and the obtained cells were centrifuged at 4°C. Red blood lysis buffer added in pelleted cells and shaked up and down 10 times, and then washed three times, finally resuspended in buffer (added 0.04% BSA in PBS). After removing dead cells in suspensions by flow cytometry, the process of single‐cell RNA‐seq following the standard instructions.

The generation of raw counts matrices was accomplished using CellRanger software, version 6.0.0, referencing the GRCm38 mouse genome. To attain a data set purified of doublets, the Scrublet software was employed. Subsequently, the filtered counts matrices underwent loading into the R software (version 4.0.3), facilitated by the Seurat package. Cells were meticulously curated to exclude those expressing either an excessive (>7000) or an insufficient (<200) number of genes, or presenting high mitochondrial counts (>20%). The raw data were then subjected to normalization using the LogNormalize method. The selection of the top 2000 highly variable genes was carried out with Seurat's FindVariableFeatures function, applying the vst method as the basis for further analysis. To harmonize the batch effects, the IntegrateData function in Seurat was employed. Dimensionality reduction was realized through Principal Component Analysis (PCA), with the leading 30 principal components (PCs) forming the basis for Uniform Manifold Approximation and Projection (UMAP) performance. Cluster identification ensued using Seurat's FindClusters function, setting the resolution at 0.5. Marker gene elucidation was achieved using the differential expression analysis through the Wilcox rank‐sum test, as implemented in the FindAllMarkers function (fold change≥2 and adjusted P≤0.05). Cell clusters underwent annotation against known cell type markers to ascertain their identity. Akin analyses focusing on subclusters of Kupffer cells and Neutrophils were conducted applying a similar methodological framework. Recognition of differentially expressed genes between two groups was facilitated by the Wilcox rank‐sum test via Seurat's FindMarkers function (fold change≥2 and adjusted P≤0.05). Data visualization was executed utilizing the ggplot2 package within the R software. Functional annotations and pathway enrichment analyses, considering the Gene Ontology (GO), were performed with the clusterProfiler package, deeming terms statistically significant at a P≤0.05.

### Tumor Treatment Experiment

C57BL/6 mice were used to construct pulmonary metastatic model. Specifically, 1×10^5^ B16F10 cells were resuspend in 0.2 mL DMEM and intravenously injected in mice. Treatments were started on day 3 after tumor inoculation. The mice were randomized allocated into four groups. The four groups were intravenously administrated with saline (control), peptide (60 µg), peptide/scramble siRNA complexes (60 µg/12 µg) or peptide/siRNA complexes (60 µg/12 µg) every other day respectively. On day 28, mice were sacrificed and lung tissues were analyzed. The number of metastatic nodules was counted in each lung. Tissues were fixed and prepared the staining with H&E and TUNEL.

For PDX model, the surgery samples of lung cancer patients were engrafted onto nude mice. Tumor fragments (about 50mm^3^) were subcutaneously engrafted into the backside of mice; the mouse generation with the grafted tumor was the P0 generation. Tumor growth was monitored weekly, and serial fragment grafts of each tumor were conducted on nude mice for the P1 generation.

### Liquid Chromatography Tandem High‐Resolution Mass Spectrometry (LC‐HRAM)

We used a developed LC‐HRAM method for the determination of peptide concentrations in mouse tissues (heart, kidney, liver, lung and spleen) and plasma. The linear range of the method was 0.200–50.0 µg ml^−1^. The heart, kidney, liver, lung and spleen tissue samples of mice were degraded into 20% tissue homogenate (tissue: homogenate solution = 1:4) with protease K. Then, 50 µl samples (standard curve samples, quality control samples, blank samples and unknown samples) were added to 10 µl of internal standard solution (500 µg ml^−1^ peptide in water) in addition to the blank samples. After vortex mixing, 100 µL of 5 mg mL^−1^ Protein K in water was added. After 1500 rpm for 1 min, multitube vortex mixing and tape winding were subsequently performed at 1000 rpm in a 65 °C constant temperature incubator overnight. For serum testing, a 10 µl sample (500 µg mL^−1^ peptide in water) of internal standard solution was added to the L sample (standard curve sample, quality control sample and blank sample). The next day, 1% FA in water and 200 µl of acetonitrile were added for protein precipitation, followed by mixing (1000 rpm, 5 min) and centrifuging (4000 rpm, 10 min). Then, 200 µl of supernatant was centrifuged at 1000 rpm for 1 min and then tested on a Shimadzu SIL‐30ACMP Autosampler with an LC‐30AD pump and a CBM‐20A controller (Shimadzu, Columbia, MD).

### Ethical Approval

The animal experiments (20190923027) and human subjects (2021619A) were approved by the Experimental Animal Management and Ethics Committee of West China Hospital, Sichuan University. All animal procedures complied with the Animal Care and Use Committee of Sichuan University.

### Statistical Analysis

Experiments were performed at least in three‐time repetition. The data are expressed as mean ± standard deviations (SDs). Comparisons between two or several groups were analyzed using unpaired student's *t*‐tests or one way analysis of variance (one‐way ANOVA, Tukey's multiple comparisons test), respectively. All statistical analyses were performed using Prism 5.0 Software (GraphPad Software, La Jolla, CA). Statistical significance was assessed by P < 0.05.

## Conflict of Interest

The authors declare no conflict of interest.

## Supporting information

Supporting Information

## Data Availability

The data that support the findings of this study are available on request from the corresponding author. The data are not publicly available due to privacy or ethical restrictions.
