# Supplementary material for: Identification of Potent siRNA Delivery Peptides Using Computer Modeling
Source: Adv Sci (Weinh). 2024 Feb 4;11(14):2308345. doi: 10.1002/advs.202308345 (PMC11005685; doi:10.1002/advs.202308345)
Supplement: Supplementary file 1 — Supporting Information [file ADVS-11-2308345-s001.pdf]

ADVANCED SCIENCE
Open Access

Supporting Information

for *Adv. Sci.*, DOI 10.1002/advs.202308345

Identification of Potent siRNA Delivery Peptides Using Computer Modeling

*Ke Men, Mohan Liu, Xueyan Zhang, Yuling Yang, Rui Zhang, Yusi Wang, Die Hu, Bailing Zhou and Li Yang**

Supporting information figures：

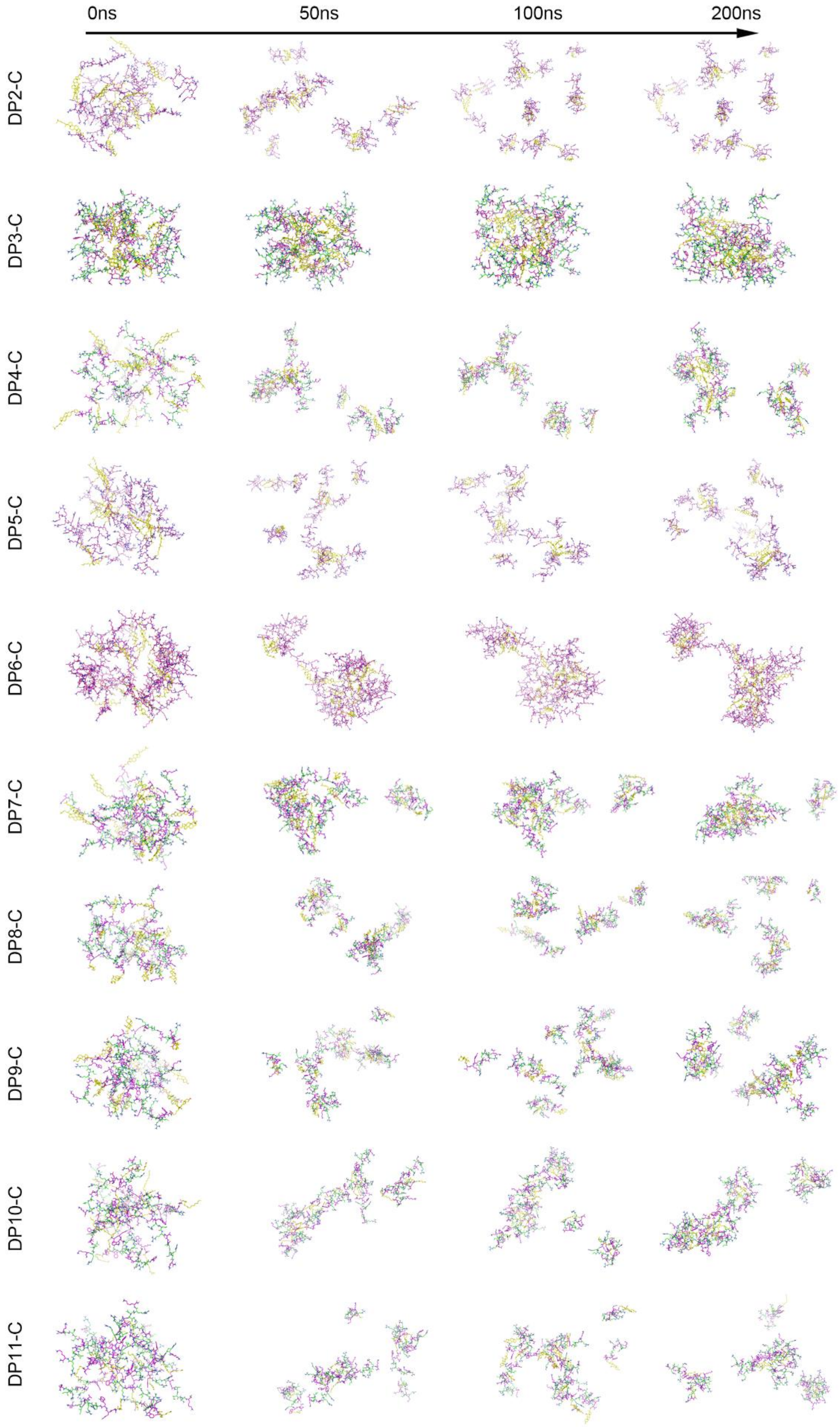

Figure S1. The structures of peptides from the library at different simulation timepoints. The carbons of cholesterol are colored yellow, and the carbons of the residues are colored blue.

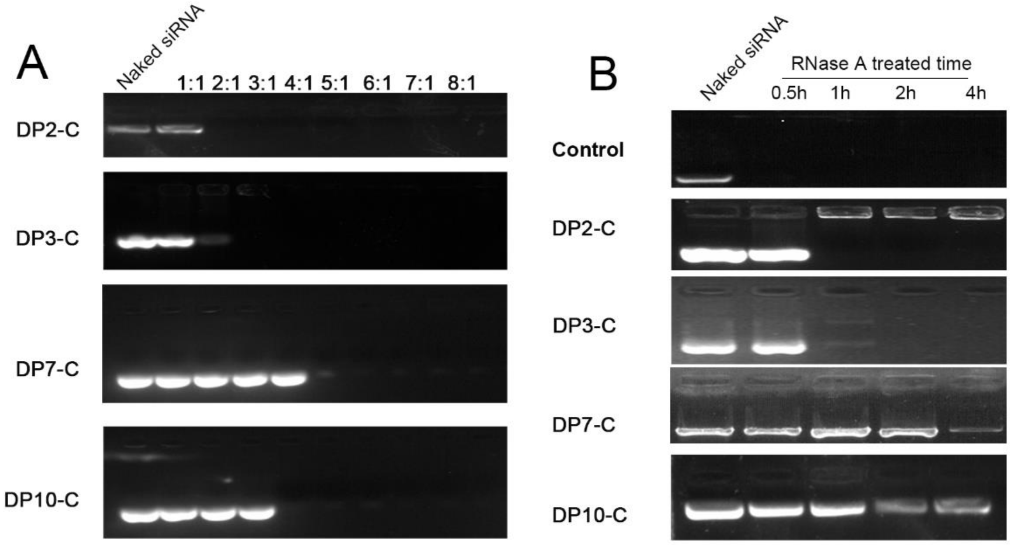

Figure S2. Characterization of peptides carrying small nucleic acids. **a,** Gel retardation analysis. **b,** Stability of small nucleic acids carried in peptides treated with RNase A *in vitro*.

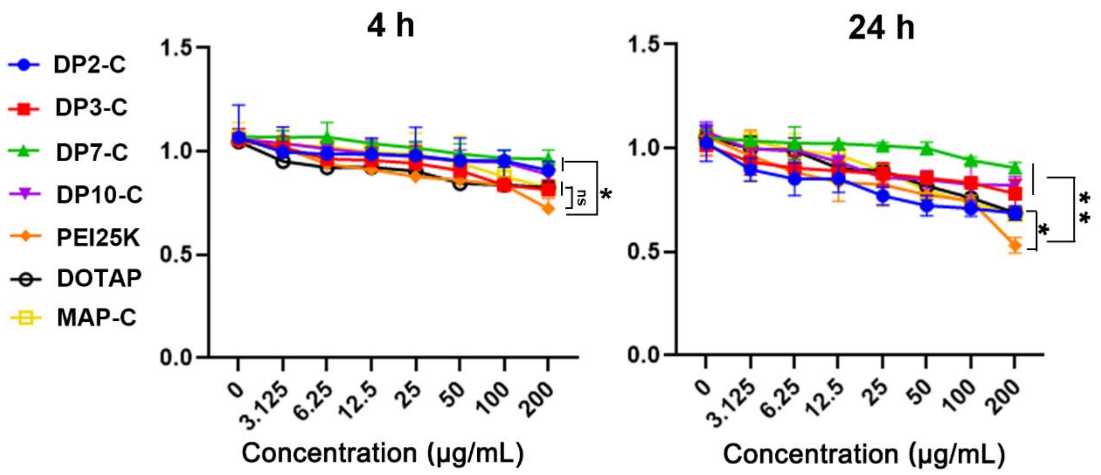

Figure S3. *In vitro* cytotoxicity of micelles toward A549 cells after 4 h and 24 h determined by MTT assay (*n*=3, data are presented as means ± SD, and the significant differences were analyzed by one-way ANOVA with Tukey's multiple comparisons test. ns, not significant; *P < 0.05; **P < 0.01).

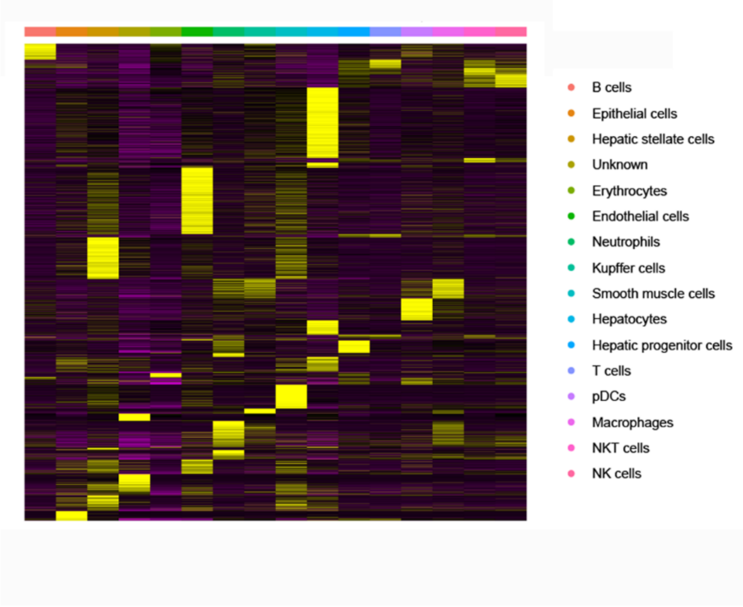

Figure S4. Heatmap showing the expression of cell type-specific marker genes in different cells in liver tissue.

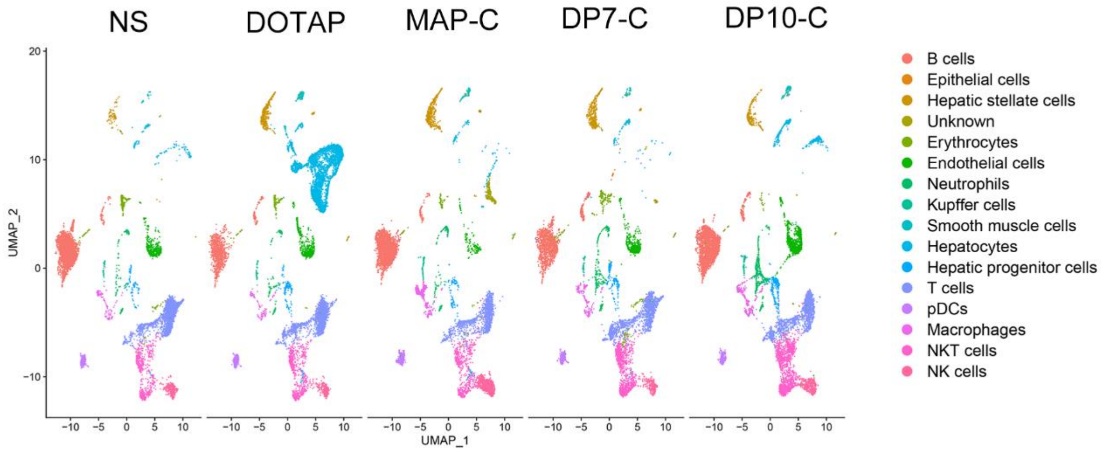

Figure S5. t-SNE map of the identified cell types in liver tissue after different administrations.

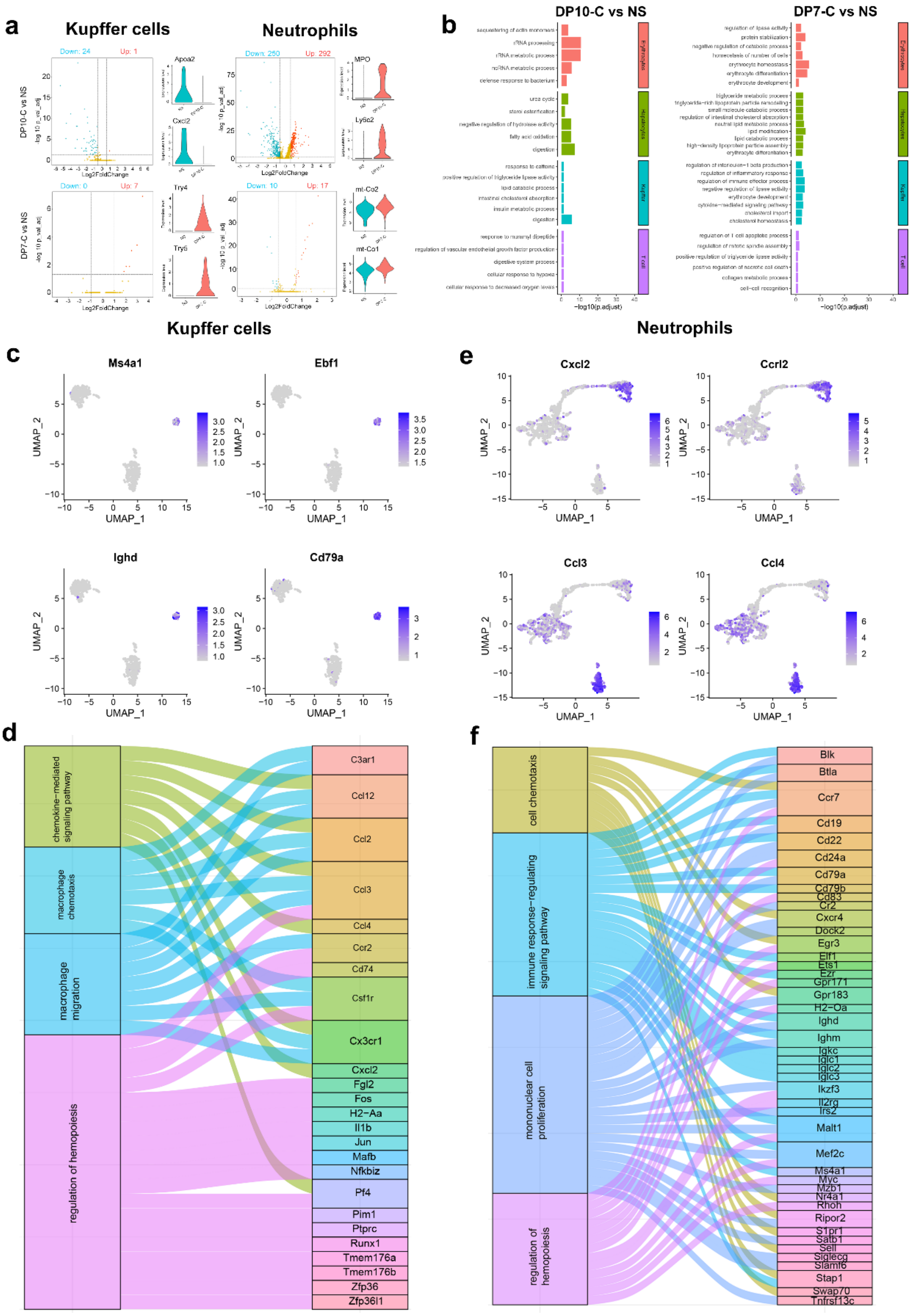

Figure S6. The specific variation in gene expression and the ratio of cells in the liver after systemic administration of DP7-C and DP10-C. **a,** Volcano plot of differential gene expression compared between the NS and peptide treatment groups. **b,** Different colored bar plots showing differences in BP functional pathways enriched per cell type

based on the DEGs. **c,** Highlighted t-SNE plot of representative Kupffer cell genes. **d,** Selected gene sets and pathways that were significantly enriched in Kupffer cell expression profiles of DP10-C-treated compared to DP7-C liver. **e,** Highlighted t-SNE plot of representative neutrophil cell genes. **f,** Selected gene sets and pathways that were significantly enriched in neutrophil expression profiles of DP10-C-treated compared to DP7-C liver.

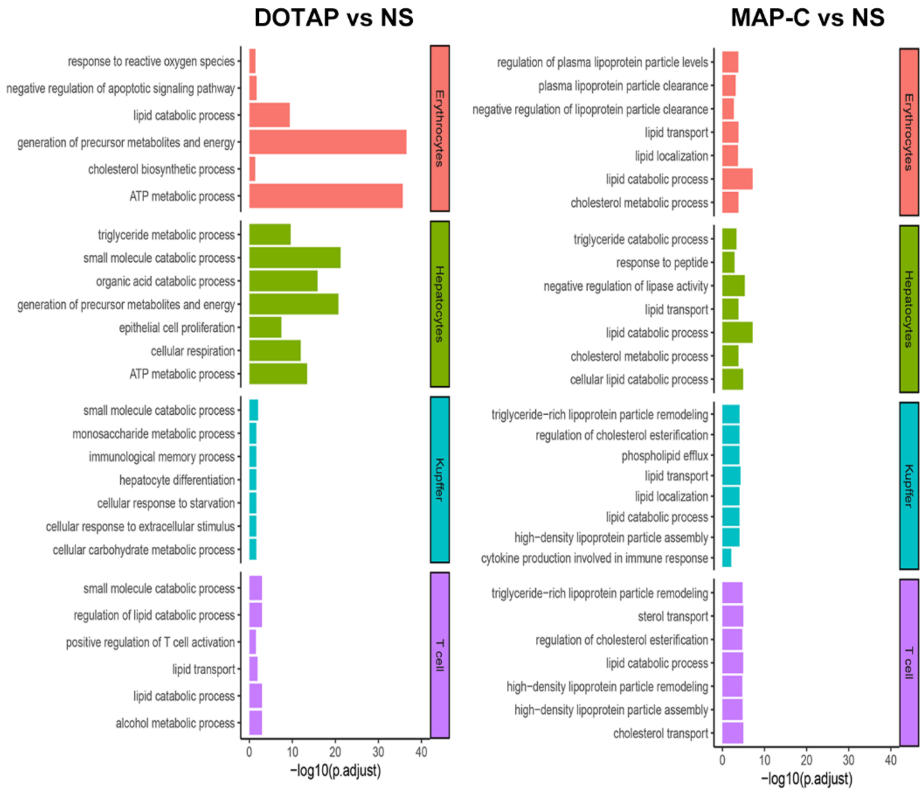

Figure S7. GO enrichment scatter plot of erythrocytes, hepatocytes, Kupffer cells and T cells in liver after different vector administrations.

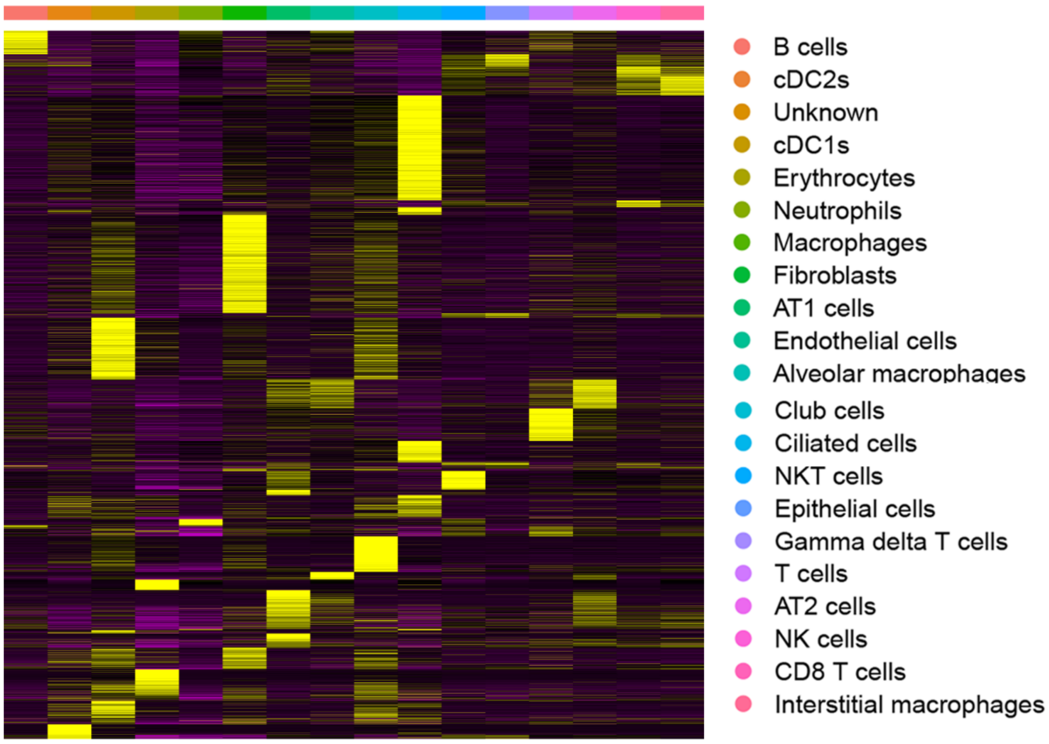

Figure S8. The expression of representative genes of various types in all lung cell clusters.

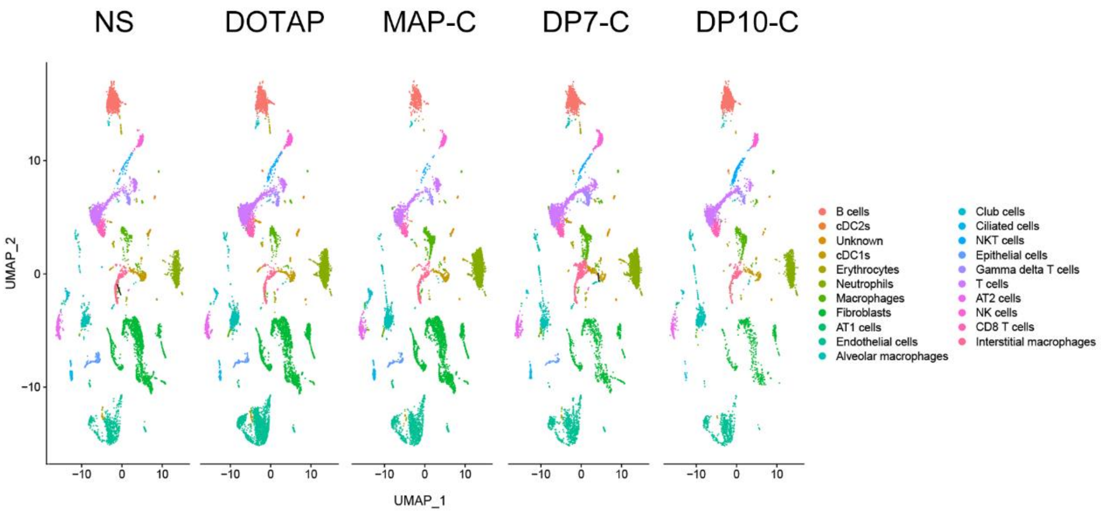

Figure S9. t-SNE plot of the identified cell types from lungs after administration of different peptides.

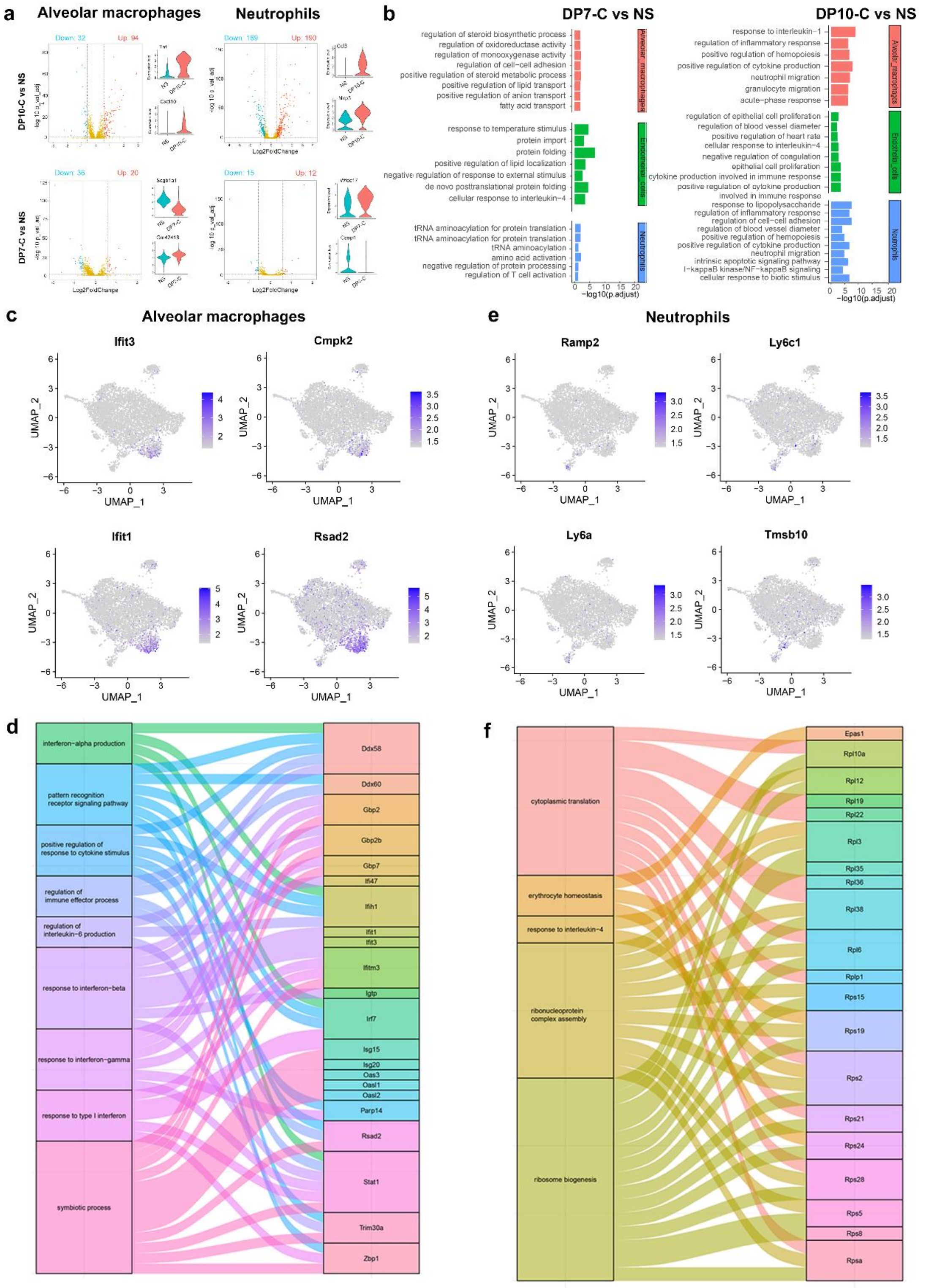

Figure S10. Specific variation in gene expression and the ratio of cells in the lungs after systemic administration of DP7-C and DP10-C. **a,** Volcano plot of differential gene expression compared between the NS and peptide treatment groups. **b,** Different colored bar plots showing differences in BP functional pathways enriched per cell type

based on the DEGs. **c,** Highlighted t-SNE plot of representative alveolar macrophage genes. **d,** Selected gene sets and pathways that were significantly enriched in alveolar macrophage expression profiles of DP10-C-treated compared to DP7-C lung. **e,** Highlighted t-SNE plot of representative neutrophil cell genes. **f,** Selected gene sets and pathways that were significantly enriched in neutrophil expression profiles of DP10-C-treated compared to DP7-C lung.

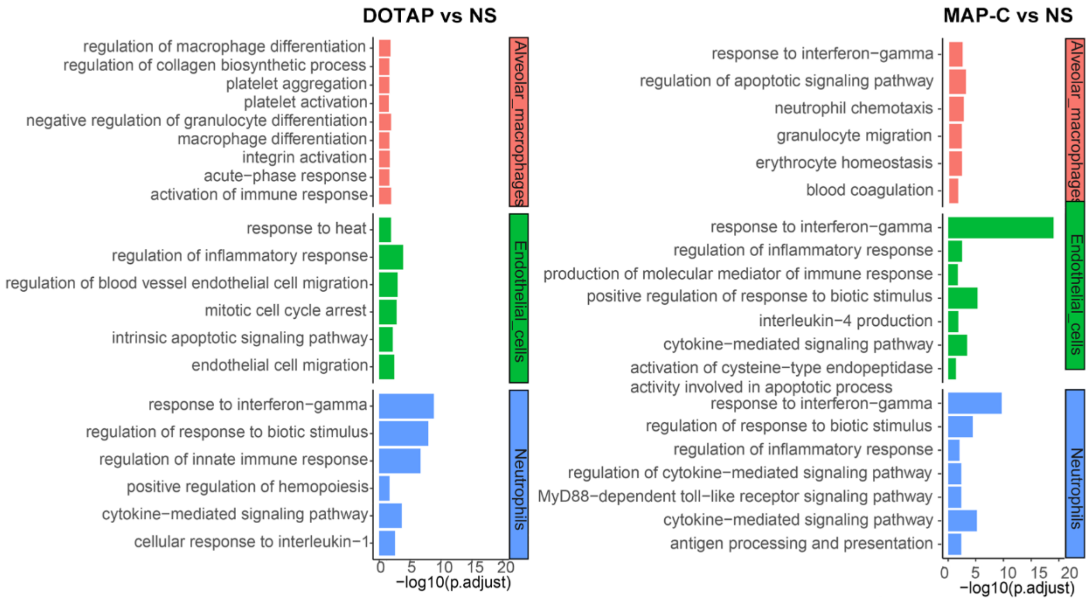

Figure S11. GO enrichment scatter plot of alveolar macrophages, endothelial cells and neutrophils in lungs.

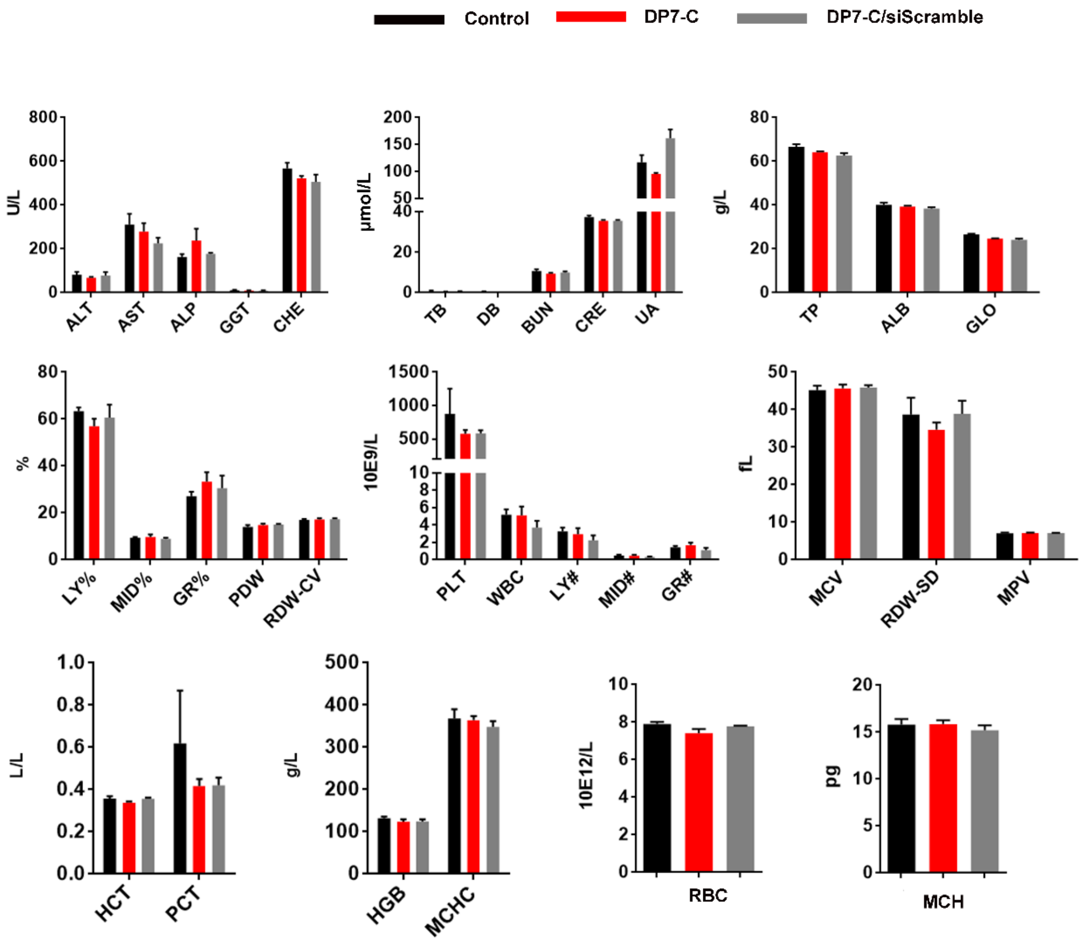

Figure S12. Toxicology assessment of C57BL/6J mice intravenously administered the DP7-C/siRNA complex. Parameters included serum-based clinical chemistry evaluation of systemic toxicity with a focus on liver and kidney function, the circulating white blood cell (WBC) count and the red blood cell (RBC) count. The dosing schedule consisted of injection of the DP7-C/siRNA (equivalent to 12 μg siRNA) complex on day 0, and blood samples were serially harvested retro-orbitally on day 0 and 24 hours after treatment. (*n*=3, data are presented as means ± SD).

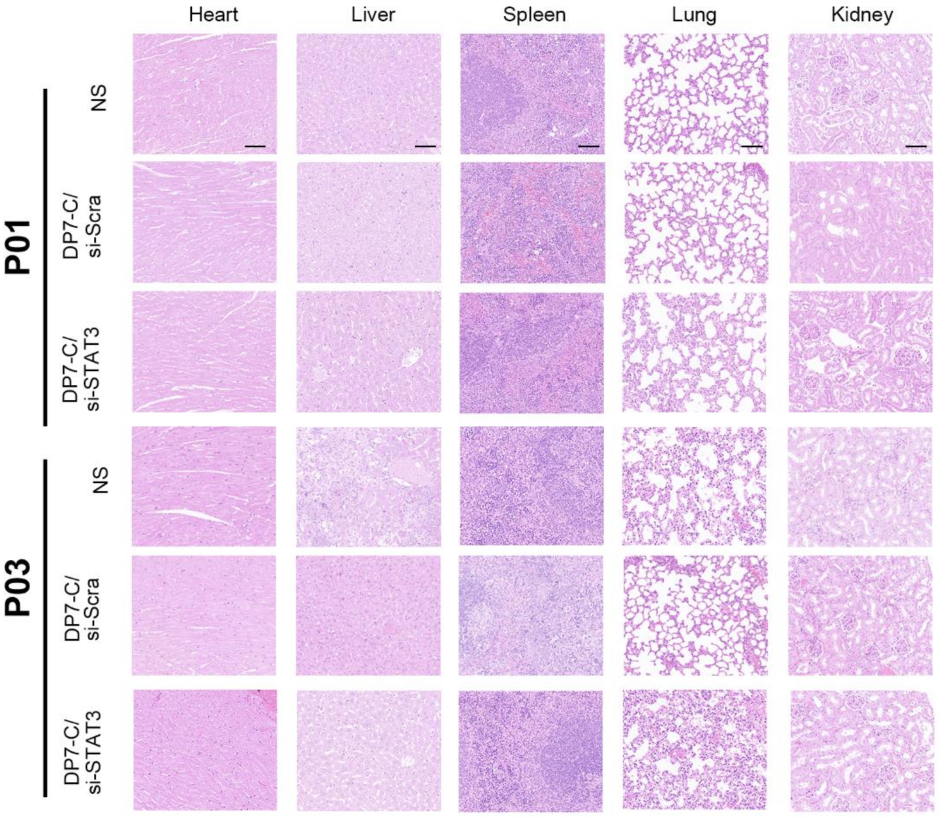

Figure S13. H&E-stained sections of mouse heart, liver, spleen, lung and kidney from the PDX model. Scale bars: 100 μm.
